# Lysine 68 Methylation‐Dependent SOX9 Stability Control Modulates Chondrogenic Differentiation in Dental Pulp Stem Cells

**DOI:** 10.1002/advs.202206757

**Published:** 2023-06-29

**Authors:** Qiannan Sun, Zimeng Zhuang, Rushui Bai, Jie Deng, Tianyi Xin, Yunfan Zhang, Qian Li, Bing Han

**Affiliations:** ^1^ Department of Orthodontics Peking University School and Hospital of Stomatology Beijing 100081 China; ^2^ National Center of Stomatology & National Clinical Research Center for Oral Diseases & National Engineering Laboratory for Digital and Material Technology of Stomatology & Beijing Key Laboratory for Digital Stomatology & Research Center of Engineering and Technology for Computerized Dentistry Ministry of Health & NMPA Key Laboratory for Dental Materials Beijing 100081 China

**Keywords:** cartilage regeneration, dental pulp stem cells, G9A, KDM3A, methylation

## Abstract

Dental pulp stem cells (DPSCs), characterized by easy availability, multi‐lineage differentiation ability, and high proliferation ability, are ideal seed cells for cartilage tissue engineering. However, the epigenetic mechanism underlying chondrogenesis in DPSCs remains elusive. Herein, it is demonstrated that KDM3A and G9A, an antagonistic pair of histone‐modifying enzymes, bidirectionally regulate the chondrogenic differentiation of DPSCs by controlling SOX9 (sex‐determining region Y‐type high‐mobility group box protein 9) degradation through lysine methylation. Transcriptomics analysis reveals that KDM3A is significantly upregulated during the chondrogenic differentiation of DPSCs. In vitro and in vivo functional analyses further indicate that KDM3A promotes chondrogenesis in DPSCs by boosting the SOX9 protein level, while G9A hinders the chondrogenic differentiation of DPSCs by reducing the SOX9 protein level. Furthermore, mechanistic studies indicate that KDM3A attenuates the ubiquitination of SOX9 by demethylating lysine (K) 68 residue, which in turn enhances SOX9 stability. Reciprocally, G9A facilitates SOX9 degradation by methylating K68 residue to increase the ubiquitination of SOX9. Meanwhile, BIX‐01294 as a highly specific G9A inhibitor significantly induces the chondrogenic differentiation of DPSCs. These findings provide a theoretical basis to ameliorate the clinical use of DPSCs in cartilage tissue‐engineering therapies.

## Graphical abstract

Antantagonistic histone‐modifying enzymes, KDM3A and G9A, bidirectionally regulate chondrogenic differentiation of dental pulp stem cells by altering the ubiquitination of non‐histone SOX9 through demethylation/methylation at K68 residue to control its degradation. Ultimately, KDM3A overexpression or G9A suppression leads to the cumulative activation of SOX9 into a chondrogenic characteristic transcriptional network both in vitro and in vivo.

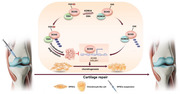

## 1 Introduction

Cartilage defects from trauma or degenerative pathology frequently associate with a disability, leading to debilitating joint pain, locking phenomena, and reduced or disturbed function.^[^ ^1^ ^]^ Unfortunately, articular cartilage has a minimal ability for self‐repair, and its regeneration remains the greatest challenging clinical problem in the field of orthopedic surgery.^[^ ^2^ ^]^ Existing clinical treatments (autologous chondrocyte implantation, osteochondral autografts, arthroplasty, etc.) have notable limitations and drawbacks, including shortage of chondrocyte source, production of nonfunctional fibrocartilage, and immune rejection, as well as limited life of the prosthesis.^[^ ^3^, ^4^, ^5^ ^]^ Recently, tissue‐engineering techniques based on mesenchymal stem cells (MSCs) are in the spotlight as a promising avenue for one‐step cartilage repair in situ.^[^ ^6^, ^7^ ^]^

Dental pulp stem cells (DPSCs) are a kind of MSCs isolated from the dental pulp of exfoliated deciduous teeth or discarded permanent teeth with minimal donor‐site morbidity and iatrogenic damage.^[^ ^8^ ^]^ The cost‐effectiveness and easy accessibility of DPSCs compared with bone marrow stem cells (BMSCs), which are usually obtained using invasive and painful methods,^[^ ^9^ ^]^ provide DPSCs with broader clinical application prospects. DPSCs originate from the neural crest and possess multi‐lineage differentiation ability to differentiate into endodermal, mesodermal, and ectodermal tissues.^[^ ^10^ ^]^ In addition, DPSCs are characterized by self‐renewal capability and higher proliferation capacity compared to BMSCs.^[^ ^11^, ^12^ ^]^ DPSCs also possess potent immunomodulatory properties that modulate the inflammatory microenvironment through Fas/FasL pathway, PD1/PD‐L1 pathway, or other immunomodulatory pathways.^[^ ^13^, ^14^, ^15^ ^]^ It has been reported that human DPSCs are able to generate cartilage‐like tissue in nude mice or repair cartilage defects in different animal models.^[^ ^16^, ^17^, ^18^ ^]^ Therefore, DPSCs are believed to be promising seed cells for cartilage tissue engineering.^[^ ^19^ ^]^ Unfortunately, the epigenetic mechanisms orchestrating DPSCs chondrogenesis remain elusive.^[^ ^20^ ^]^ Therefore, it appears quite imperative to uncover the critical regulators to ameliorate the chondrogenic efficiency of DPSCs.

MSCs chondrogenesis is coordinated by a series of signaling pathways and transcription factors (TFs). Among them, the master transcription factor SOX9 (sex‐determining region Y‐type high‐mobility group box protein 9) plays a key role in the chondrogenic differentiation of MSCs by directly binding to the promoter or enhancer region of certain chondrocyte matrix genes, such as aggrecan (ACAN), cartilage oligomeric matrix protein (COMP), and type II collagen alpha 1 chain (COL2A1), thereby activating their expression.^[^ ^21^ ^]^ Epigenetic factors governing histone modifications, including histone methylation and acetylation, have been proven to be important regulators of SOX9 transcriptional level or activity.^[^ ^22^ ^]^ In addition to histone modifications, post‐translational modifications of non‐histone proteins are important epigenetic regulatory mechanisms and have been implicated in differential biological events. In recent years, research on the post‐translational regulation of SOX9 during chondrogenesis has emerged. The major histone deacetylase SIRT1 deacetylates SOX9 to increase its nuclear localization and transactivation of target genes,^[^ ^23^ ^]^ whereas Tat‐interacting protein 60 acetylates SOX9 through multiple lysine residues to enhance its transactivation.^[^ ^24^ ^]^ Phosphorylation of SOX9 by catalytic subunit of protein kinase A enhances its transactivation activity.^[^ ^25^ ^]^ Furthermore, the E3 ubiquitin ligase UBE3A is identified as a ubiquitin ligase for SOX9 in chondrocytes.^[^ ^26^ ^]^ Protein arginine methyltransferases PRMT4 and PRMT5 have been reported to be involved in the arginine methylation of SOX9.^[^ ^27^, ^28^ ^]^ However, to date, the molecular mechanisms regarding post‐translational lysine methylation of SOX9 during chondrogenesis have not been identified.

Lysine demethylase 3A (KDM3A) is a member of the histone demethylase family. With a catalytic Jumonji C (JmjC) domain, KDM3A catalyzes the demethylation of mono‐ and di‐methylated histone H3 lysine 9 (H3K9me1/me2), thereby mediating transcriptional activation.^[^ ^29^ ^]^ The methyltransferase G9A, also called EHMT2, forms heterodimers with G9A‐like protein (GLP, also known as EHMT1) and methylates H3K9me1 and H3K9me2 (as opposed to KDM3A).^[^ ^30^ ^]^ In addition to histone H3, some non‐histone proteins were found to be substrates of KDM3A and G9A. For example, KDM3A was revealed to mediate lysine demethylation of peroxisome proliferator‐activated receptor gamma coactivator‐1 alpha, an important regulator of mitochondrial biogenesis in response to oxygen availability.^[^ ^31^ ^]^ KDM3A was also shown to regulate breast cancer cell apoptosis by demethylating the tumor suppressor p53.^[^ ^32^ ^]^ Meanwhile, G9A was found to mediate lysine methylation of p53, CCAAT/enhancer bingding protein beta, and forkhead box transcription factor O1 (FOXO1).^[^ ^33^, ^34^, ^35^ ^]^ Mounting evidence suggests the critical regulatory role of KDM3A and G9A in diverse biological events such as metabolism,^[^ ^29^ ^]^ cancer progression,^[^ ^36^ ^]^ spermatogenesis,^[^ ^37^ ^]^ stem cell function,^[^ ^38^ ^]^ and sex determination.^[^ ^39^ ^]^ In BMSCs, KDM3A and G9A play an essential role in osteogenesis through their classical histone demethylation or methylation activity.^[^ ^40^, ^41^ ^]^ KDM3A also regulates mesenchymal stromal cell senescence and bone aging via condensin‐mediated heterochromatin reorganization,^[^ ^42^ ^]^ but whether they are involved in the chondrogenic differentiation of MSCs remains a mystery.

**Figure 1:**
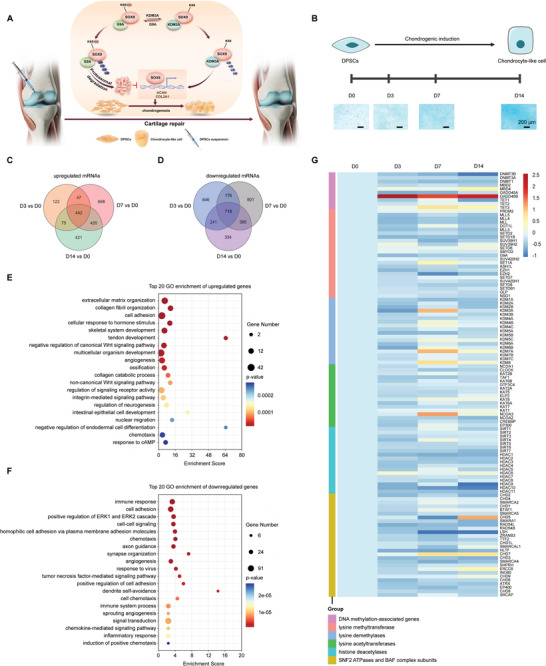
Graphical illustration of the whole study and transcriptomic analysis of DPSCs during chondrogenic differentiation. A) Schematic illustration of the mechanism by which DPSCs promote the in situ repair of cartilage defects in rat knees. KDM3A promotes chondrogenic differentiation by elevating SOX9 stability through lysine demethylation, whereas G9A‐dependent lysine methylation counteracted this process. B) Schematic overview of morphological changes in DPSCs during chondrogenic differentiation (upper) and alcian blue staining of the chondrogenic differentiation process (lower). Scale bars: 200 µm. C,D) Venn diagrams showing overlapping relationships of upregulated and downregulated genes in three comparison groups. E) GO enrichment analysis of overlapped DEGs of three comparison groups as in (C). F) GO enrichment analysis of overlapped DEGs of three comparison groups as in (D). 20 pathways were arranged from top to bottom according to *Q* values. G) Heatmap showing the dynamic expression (log2 transformed FPKM, fragments per kilobase of transcript per million fragments mapped) of epigenetic regulators during DPSCs chondrogenesis. *n* = 3 biological replicates. D, Day; GO, Gene ontology.

Here, we identify KDM3A as a positive regulator of chondrogenesis in DPSCs. More importantly, KDM3A increases the abundance of SOX9 protein by antagonizing the methyltransferase G9A. Mechanistically, KDM3A enhances SOX9 stability by demethylating lysine (K) 68 residue to attenuate the ubiquitination of SOX9, whereas G9A counteracts this process through lysine methylation of SOX9 at K68 residue (**Figure** **1**A). Furthermore, BIX‐01294 as a specific G9A inhibitor significantly promotes the chondrogenic differentiation of DPSCs, providing a good foreshadowing for the future translation of clinical applications.

## 2 Results

### 2.1 Transcriptomic Analysis of mRNA Changes During Chondrogenic Differentiation of DPSCs

To characterize the immunophenotype of obtained DPSCs, flow cytometry analysis was performed. Results showed that DPSCs were positive for stem cell surface markers CD105, CD90, and CD73, but were negative for hematopoietic lineage markers CD45 (Figure S1, Supporting Information). The phenotypes matched the criteria for MSCs identification.^[^ ^43^ ^]^ To screen for vital regulators during DPSCs chondrogenesis, it is necessary to first examine global genetic changes in the process of chondrogenic differentiation of DPSCs. To this end, DPSCs were cultured in a chondrogenic induction medium for 0, 3, 7, or 14 days (D0, D3, D7, or D14), respectively, and mRNAs were extracted at the indicated time points (Figure 1B). Then high‐throughput RNA‐sequencing (RNA‐seq) was conducted to profile the genome‐wide mRNA expression patterns. Comparative analyses were performed between each two groups and the numbers of upregulated and downregulated differentially expressed genes (DEGs) are listed in Figure S2, Supporting Information. We noticed that D7 versus D0 had the most DEGs, with 1607 upregulated and 1790 downregulated genes. Venn analyses were further performed to visualize the shared or unique genes among the three comparison groups (D3 vs D0, D7 vs D0, and D14 vs D0). Overall, 442 genes were simultaneously upregulated (Figure 1C), whereas 718 genes were downregulated on D3, D7, and D14 (Figure 1D).

To gain insight into the biological roles of the DEGs, a gene ontology (GO) analysis of these overlapping genes was performed. Results showed that the shared upregulated genes were mostly enriched in the extracellular matrix (ECM) organization, collagen fibril organization, and cell adhesion (Figure 1E). The shared downregulated genes were implicated in the regulation of immune response, cell adhesion, positive regulation of ERK1 and ERK2 cascade, etc (Figure 1F). Kyoto Encyclopedia of Genes and Genomes (KEGG) pathway analysis identified significantly enriched pathways in DEGs, including cytokine–cytokine receptor interaction, protein digestion, and ECM‐receptor interaction, among others (Figure S3, Supporting Information).

Subsequently, to identify key epigenetic factors that determine the chondrogenesis of DPSCs, changes in the expression of epigenetic genes belonging to six subgroups were analyzed and the results are shown as heatmaps in Figure 1G. In DNA‐methylation‐associated genes, decreased expression of DNMT3B and TET1 was observed at D14, but GADD45B showed robust upregulation since D3. The expression of lysine methyltransferases exhibited a general decline upon chondrogenic induction with EZH2 significantly downregulated at D7. In contrast, lysine demethylases KDM3A, −7A, and −8 showed marked increases. Most histone acetylation regulators showed marginal alterations, with upregulation of NCOA3 and downregulation of HDAC9 and HDAC10. In chromatin‐remodeling associated genes, it was noticed that CHD5 and −7 showed elevated expression at D14, while LSH dropped to a low level since D7. We speculated that these epigenetic modulators might be involved in DPSCs chondrogenesis.

### 2.2 The Expression of KDM3A Increases Along with Chondrogenic Differentiation in Response to TGF*β* Signaling

**Figure 2:**
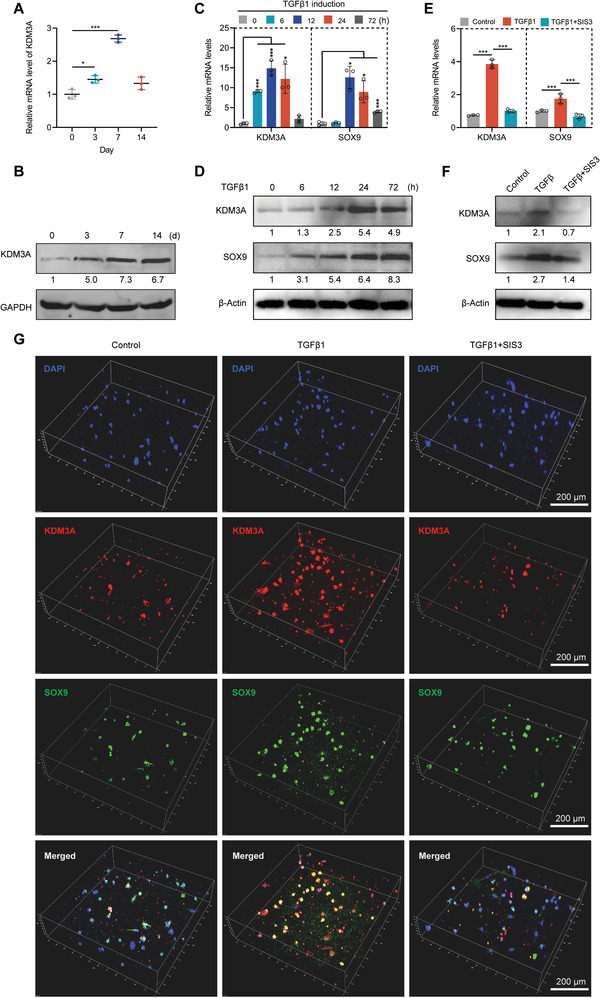
TGF*β*1 promotes the expression and activity of KDM3A through TGF*β*1/SMAD signaling. A) RT‐qPCR showing the mRNA level of KDM3A during chondrogenic differentiation of DPSCs. B) Western blotting showing the total protein level of KDM3A during chondrogenic differentiation of DPSCs. RT‐qPCR (C) and western blotting (D) showing the expression levels of KDM3A and SOX9 after incubation with TGF*β*1 (10 ng mL^−1^) for 0, 6, 12, 24, or 72 h. E,F) The expression levels of KDM3A and SOX9 after incubation with TGF*β*1 (10 ng mL^−1^) or TGF*β*1 (10 ng mL^−1^) and SIS3 (3 µm) for 24 h, as determined by RT‐qPCR (E) and western blotting (F). Untreated DPSCs were used as control. G) Immunofluorescence staining revealing the expression levels of KDM3A and SOX9 in 3D cultured DPSCs after incubation with TGF*β*1 (10 ng mL^−1^) or TGF*β*1 (10 ng mL^−1^) and SIS3 (3 µm) for 24 h. Scale bars: 200 µm. The relative densities of proteins indicated below the blots were first normalized to that of internal reference proteins and then calculated as a ratio relative to the value in control cells. Each bar represents mean ± SD; *n* = 3 per group; *p < 0.05, ***p < 0.001.

To search for a key epigenetic modulator for further investigation, reverse transcription–quantitative polymerase chain reaction (RT‐qPCR) was then performed to validate the expression changes based on RNA‐seq analysis (Figure 1G). GADD45B with the most significant change in RNA‐seq analysis was first validated. RT‐qPCR showed that the expression of GADD45B was significantly upregulated during the chondrogenic differentiation of DPSCs. However, siRNA‐mediated knockdown of GADD45B led to no significant change in the chondrogenic differentiation capacity of DPSCs as shown by alcian blue staining, indicating that transcriptional upregulation of GADD45B might be a result rather than an indispensable cause of DPSCs' chondrogenesis (Figure S4, Supporting Information). Therefore, GADD45B was not further explored as the main object. Moreover, methyltransferases and histone demethylases are of particular interest due to their prominent roles in stem cell fate decisions and the relative experience gained from our previous work. Through RT‐qPCR, we found that KDM3A was stable and upregulated upon chondrogenic induction (**Figure** **2**A), in parallel with the expression pattern in RNA‐seq analysis. Western blotting revealed a significant increase of KDM3A at the protein level (Figure 2B), indicating that KDM3A expression was induced at both the mRNA and protein levels along with chondrogenic differentiation of DPSCs.

Transforming growth factor‐beta1 (TGF*β*1) is one of the important factors involved in chondrogenic differentiation, and TGF*β*1/SMAD signaling promotes the expression of SOX9, COL2A1, and ACAN genes.^[^ ^44^ ^]^ Moreover, it has been demonstrated that KDM3A can respond to TGF*β* signaling in cardiac myofibroblast transdifferentiation and smooth muscle cell differentiation.^[^ ^45^, ^46^ ^]^ Hence, we then investigated the implications of TGF*β*1 on the expression of KDM3A during chondrogenic differentiation. The expression of SOX9 was used as a positive control to validate the effectiveness of TGF*β*1/SMAD signaling activation. RT‐qPCR showed simultaneously enhanced expression of KDM3A and SOX9 with prolongation of TGF*β*1 treatment time, and the expression levels peaked at 12 h of treatment (Figure 2C). Western blotting revealed that KDM3A and SOX9 protein levels significantly increased after incubation with TGF*β*1 (Figure 2D). TGF*β*1/SMAD signaling is the canonical TGF*β*1 pathway. Hence, we utilized SIS3, a chemical inhibitor of SMAD3, to determine whether SMAD3 is essential for the TGF*β*1‐induced upregulation of KDM3A. After incubation with SIS3 for 24 h, the increased levels of KDM3A and SOX9 mRNA and protein triggered by TGF*β*1 were successfully suppressed (Figure 2E,F). To create more realistic biochemical and biomechanical microenvironments, a 3D cell culture system based on Matrigel was adopted. Immunofluorescence analysis confirmed that TGF*β*1 simultaneously enhanced the expression of KDM3A and SOX9, whereas SIS3 greatly suppressed the TGF*β*1‐induced expression of KDM3A and SOX9 (Figure 2G). These results suggest that KDM3A expression increases in response to the upstream TGF*β*1 signaling during chondrogenesis.

### 2.3 KDM3A is Required for the Chondrogenic Differentiation of DPSCs

**Figure 3:**
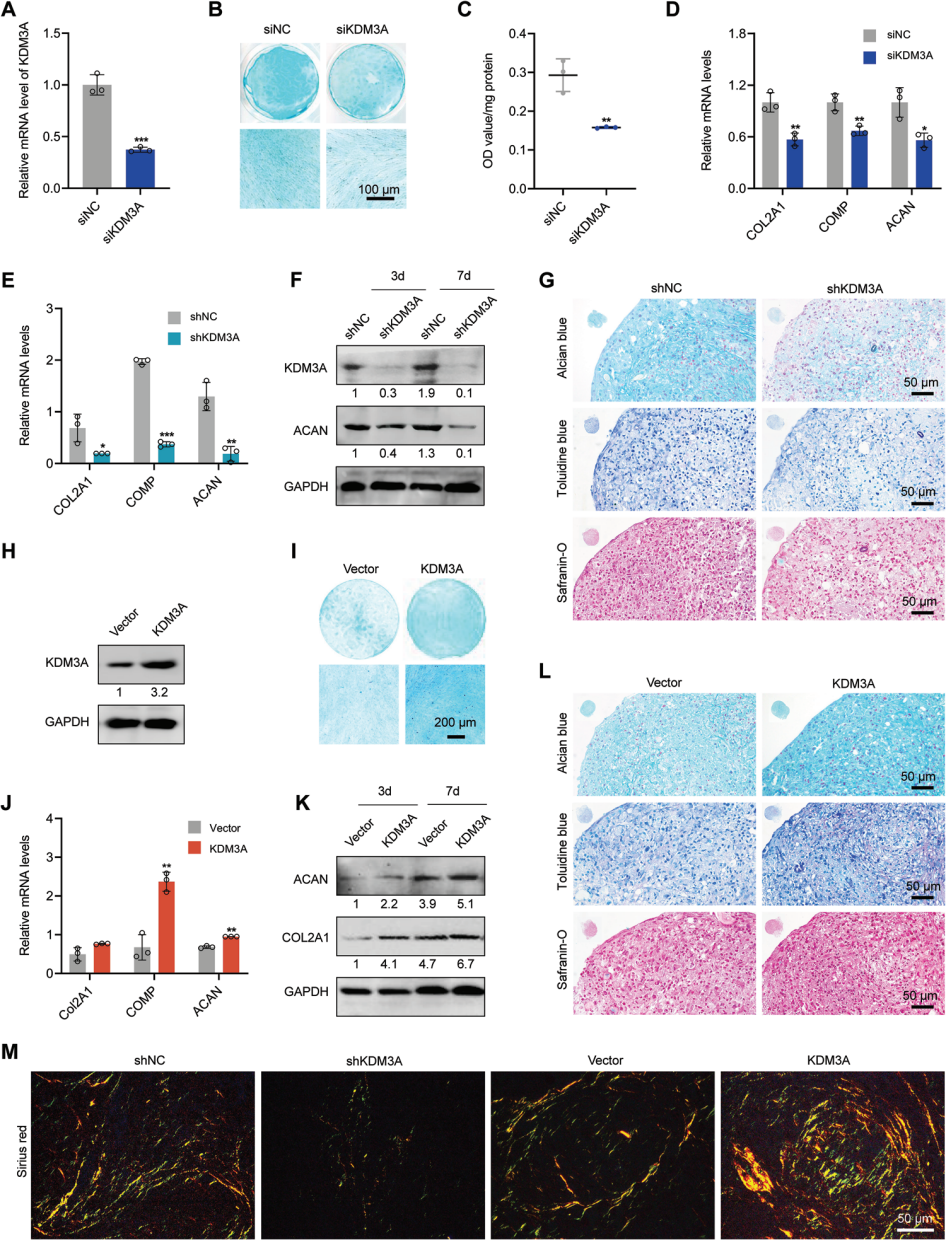
KDM3A is required for the chondrogenic differentiation of DPSCs. A) RT‐qPCR analysis showing the knockdown efficiency of siRNA against KDM3A (siKDM3A). B) Alcian blue staining revealing KDM3A knockdown effectively restrained proteoglycan production after induction with chondrogenic medium for 2 weeks. Scale bar: 100 µm. C) Quantification of proteoglycans synthesis at 2 weeks. D) Expression levels of indicated cartilage‐signature markers were determined in DPSCs induced with chondrogenic differentiation media for 7 days. DPSCs were transfected with lentivirus‐mediated shRNA targeting KDM3A (shKDM3A) or negative control (shNC). Expression levels of indicated genes were performed by RT‐qPCR (E) and western blotting (F). G) DPSC pellets transfected with indicated expression constructs were stained with alcian blue, toluidine blue, and safranin‐O after a 2‐week chondrogenic induction. Scale bars: 50 µm. H) Western blotting showing the enhanced expression of KDM3A after DPSCs transduced with lentiviral‐KDM3A. I) Alcian blue staining revealing KDM3A overexpression effectively elevated proteoglycan production after being induced with a chondrogenic medium for 2 weeks. Scale bar: 200 µm. RT‐qPCR (J) and western blotting (K) indicating increased expression of cartilage‐signature markers in KDM3A‐overexpressed DPSCs. L) Alcian blue, toluidine blue, and safranin‐O indicating augmented cartilage matrix in KDM3A‐overexpressed pellets. Scale bars: 50 µm. M) Sirius red showing collagen formation of DPSC pellets transfected with indicated expression constructs after a 2‐week chondrogenic induction. Scale bars: 50 µm. The relative densities of proteins indicated below the blots were first normalized to that of internal reference proteins and then calculated as a ratio relative to the value in control cells. Each bar represents mean ± SD; *n* = 3 per group; *p < 0.05, **p < 0.01, ***p < 0.001.

To examine the potential regulatory role of KDM3A in cartilage formation, small interfering RNA (siRNA) against KDM3A (siKDM3A) and the scrambled control siRNA (siNC) was transfected into DPSCs. RT‐qPCR indicated that the knockdown efficiency of KDM3A was ≈60% compared to that in the corresponding siNC group (**Figure** **3**A). Alcian blue staining assay revealed that proteoglycan production was significantly decreased in KDM3A‐depleted DPSCs after chondrogenic induction for 2 weeks (Figure 3B,C). We further examined the expression of cartilage‐signature markers, including COL2A1, COMP, and ACAN, by RT‐qPCR. Results showed that KDM3A knockdown significantly suppressed the mRNA levels of all three markers after chondrogenic induction for 1 week (Figure 3D). Consistently, small hairpin RNA (shRNA)‐mediated KDM3A (shKDM3A) depletion also resulted in inhibited chondrogenic differentiation, as demonstrated by the downregulation of cartilage‐signature genes (Figure 3E,F). The chondrogenic differentiation of MSCs has been reported to be more efficient in micro‐mass culture. Therefore, to further consolidate our conclusion, DPSCs were infected with lentivirus expressing shRNA of KDM3A followed by chondrogenic induction in a pellet culture system. Alcian blue, toluidine blue, and safranin O staining revealed the distribution and content of proteoglycans produced by cells. Results indicated that KDM3A depletion significantly impeded the production of proteoglycans (Figure 3G).

To comprehensively elucidate the effect of KDM3A overexpression on DPSCs chondrogenesis, DPSCs transduced with lentiviral KDM3A were subjected to subsequent experiments. As demonstrated by immunoblot, KDM3A protein was greatly increased by lentivirus‐mediated overexpression (Figure 3H). Then, KDM3A‐overexpressed DPSCs were cultured in a chondrogenic induction medium for 2 weeks, resulting in a notable augmentation in proteoglycans (Figure 3I). Additionally, RT‐qPCR and western blotting revealed increased expression of cartilage‐signature genes in KDM3A‐overexpressed cells (Figure 3J,K). Similar to monolayered DPSCs, KDM3A overexpression resulted in increased proteoglycan production in DPSC pellets (Figure 3L). Consistently, collagen was markedly augmented in KDM3A overexpression pellets but was barely detectable in KDM3A silencing pellets (Figure 3M and Figure S5, Supporting Information). Thus, KDM3A is an essential regulator of chondrogenesis and strengthens the overall chondrogenic features in DPSCs.

### 2.4 KDM3A Regulates SOX9 Expression at Protein Level by Antagonizing G9A

**Figure 4:**
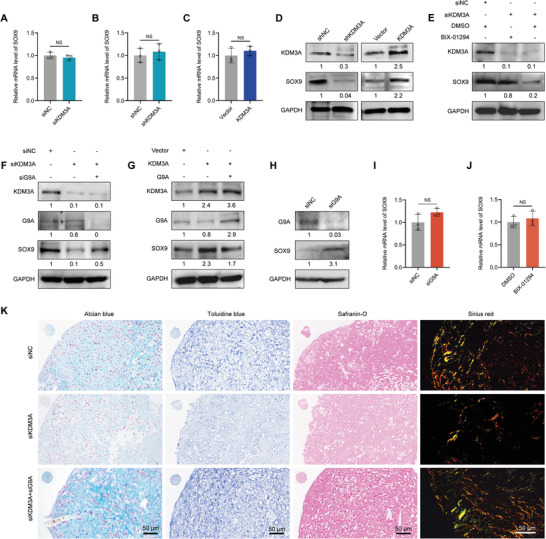
KDM3A regulates SOX9 expression at the protein level in a G9A‐dependent manner. A–C) RT‐qPCR analysis showing mRNA level of SOX9 after DPSCs transfected with indicated expression constructs. D) Western blotting indicating changes in SOX9 protein level after KDM3A depletion or overexpression in DPSCs. E) Western blotting indicating the downregulation of SOX9 protein after KDM3A knockdown, and ameliorated SOX9 protein level after treatment with BIX‐01294 (2 µm). F) Western blotting showing that G9A knockdown could improve the decreased SOX9 protein level caused by KDM3A depletion. G) Western blotting showing that G9A overexpression could inhibit the increased SOX9 protein level caused by KDM3A overexpression. H) Western blotting showing enhanced SOX9 protein level after G9A knockdown. I,J) RT‐qPCR analysis showing mRNA level of SOX9 in DPSCs transfected with siRNA against G9A (I) or treated with drug (J). K) Alcian blue, toluidine blue, safranin‐O, and sirius red indicating proteoglycan production and collagen formation in DPSC pellets transfected with indicated expression constructs after a 2‐week chondrogenic induction. Scale bar: 50 µm. The relative densities of proteins indicated below the blots were first normalized to that of internal reference proteins and then calculated as a ratio relative to the value in control cells. Each bar represents mean ± SD; *n* = 3 per group; NS: not significant.

Having established the essential role of KDM3A in the chondrogenic differentiation of DPSCs, then we investigated the underlying molecular mechanism. Flow cytometry analysis and RT‐qPCR showed that KDM3A knockdown had no significant effect on the stem cell surface markers or stemness properties of DPSCs (Figures S1 and S6, Supporting Information). Given that SOX9 is the master transcription factor in chondrogenic differentiation, we investigated whether SOX9 expression is influenced by KDM3A. RT‐qPCR revealed that the mRNA level of SOX9 was nearly unchanged when KDM3A was knocked down (**Figure** **4**A,B). Meanwhile, SOX9 mRNA level was unaffected by KDM3A overexpression (Figure 4C). However, the SOX9 protein level was markedly reduced upon shRNA‐mediated depletion of KDM3A (Figure 4D). In contrast, ectopic expression of KDM3A significantly stimulated SOX9 protein expression in DPSCs (Figure 4D). Similar results were obtained from human bone marrow mesenchymal stem cells (hBMSCs, Figure S7, Supporting Information). These results suggest that KDM3A positively regulates SOX9 at the protein level. G9A is a lysine methyltransferase that acts in opposition to the KDM family demethylases on histone H3 lysine 9 (H3K9) in the modulation of the expression of several genes.^[^ ^47^, ^48^ ^]^ Therefore, we next tested the possibility of G9A participating in SOX9 regulation. As shown by flow cytometry analysis and RT‐qPCR, G9A knockdown had no effect on surface markers or stemness markers expression in DPSCs (Figures S1 and S6, Supporting Information). Intriguingly, we noticed that the downregulation of SOX9 protein level caused by KDM3A depletion was partially rescued by the treatment of BIX‐01294, a specific small‐molecule inhibitor of G9A (Figure 4E). Consistently, cells with simultaneous knockdown of KDM3A and G9A exhibited higher protein levels of SOX9 than those with individual depletion of KDM3A (Figure 4F), indicating that KDM3A and G9A likely modulate SOX9 expression by antagonizing each other. We then investigated the effect of G9A on SOX9 expression in the case of KDM3A overexpression. Results showed that G9A overexpression suppressed the elevated SOX9 protein level caused by KDM3A overexpression (Figure 4G). Moreover, siRNA‐mediated knockdown of G9A led to elevated SOX9 expression at the protein level without any impact on the mRNA level (Figure 4H,I and Figure S8, Supporting Information). BIX‐01294 also led to an unaltered mRNA level of SOX9 (Figure 4J), implying that G9A‐dependent SOX9 regulation was also at the post‐translational level.

These results underscore the post‐translational regulation of SOX9 mediated by KDM3A and G9A in a mutually antagonistic manner. To verify whether G9A influences the chondrogenic differentiation capacity of DPSCs as opposed to KDM3A, we used the aforementioned pellet culture system to induce chondrogenesis. Similar to the shRNA‐mediated knockdown of KDM3A, DPSCs transfected with siRNA against KDM3A also showed weakened chondrogenesis (Figure 4K and Figure S9, Supporting Information). Conversely, G9A knockdown by siRNA led to strengthened chondrogenesis of DPSCs, as revealed by increased proteoglycan and collagen formation, indicating that G9A silencing enhanced the chondrogenic potential of DPSCs, in accordance with the upregulated SOX9 protein level upon G9A depletion.

### 2.5 KDM3A and G9A Regulate the Lysine Methylation Level of SOX9

**Figure 5:**
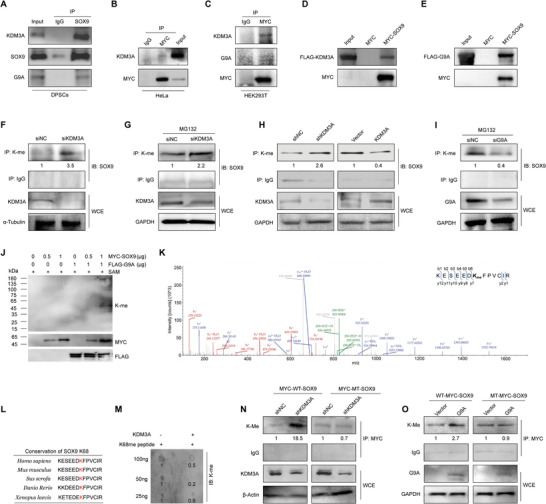
KDM3A and G9A regulate the lysine methylation level of SOX9. A) Protein extracts from DPSCs were treated with RNase A (10 µg mL^−1^), and then subjected to co‐immunoprecipitation experiments with SOX9 antibody used for immunoprecipitation and indicated antibodies used for immunoblotting. B,C) Protein extracts from HeLa or HEK293T cells were subjected to co‐immunoprecipitation experiments with MYC antibody used for immunoprecipitation and indicated antibodies used for immunoblotting. D,E) Pull‐down experiments were performed with bacterially purified MYC‐SOX9 and in vitro transcribed/translated FLAG‐KDM3A and FLAG‐G9A. F–I) Proteins were extracted from DPSCs (transfected with indicated molecules) and immunoprecipitation experiments were then conducted with IgG and K‐me antibodies used for immunoprecipitation and indicated antibodies used for immunoblotting. DPSCs used in (G) and (I) were treated with MG132 (20 µm) for 5 h before harvesting. J) Western blotting indicating the effect of G9A on the methylation level of SOX9 in vitro. S‐adenosylmethionine (SAM) as a methyl donor. The standard molecular mass (in kDa) of proteins is indicated on the left. K) Mass spectrometry revealing the methylation site of SOX9 that G9A binds to. L) Amino acid sequence of K68me showing high conservation among vertebrates. M) Dot blot assay showing the demethylation effect of KDM3A on the K68me peptide of SOX9. N,O) MYC‐tagged mutant (MT) SOX9 or MYC‐tagged wild‐type (WT) SOX9 and lentivirus‐mediated shRNA targeting KDM3A (shKDM3A), lentiviral‐G9A or the control vector were co‐transfected into HEK293T cells. Immunoprecipitation experiments were then performed with MYC antibodies used for immunoprecipitation and indicated antibodies used for immunoblotting. The relative densities of proteins indicated below the blots were first normalized to that of internal reference proteins and then calculated as a ratio relative to the value in control cells. kDa: kilodalton; IP: immunoprecipitation; IB: immunoblotting; WCE: whole cell lysate; K‐me: lysine methylation.

To further explore the post‐translational regulatory mechanism exerted by KDM3A and G9A, it was necessary to determine whether they physically interacted with SOX9. We co‐immunoprecipitated endogenous SOX9 from DPSC lysates and performed western blotting. Results indicated that KDM3A and G9A efficiently precipitated along with SOX9 (**Figure** **5**A), suggesting an in vivo association between them. To clarify whether this interaction also exists in other types of cells, the plasmid encoding MYC‐tagged SOX9 (MYC‐SOX9) was transfected into HeLa and human embryonic kidney 293T (HEK293T) cells, respectively, followed by co‐immunoprecipitation assay. As shown by western blotting, MYC antibody as a bait successfully precipitated KDM3A in HeLa and HEK293T cells, as well as G9A in HEK293T cells (Figure 5B,C). Hence, the interaction among KDM3A, G9A, and SOX9 may be a universal phenomenon. Next, a pull‐down assay was performed to determine whether the interaction was direct or indirect. In vitro purified MYC‐SOX9 or MYC peptides were first loaded onto MYC‐magnetic beads, and then FLAG‐KDM3A or FLAG‐G9A proteins were incubated together. Western blotting showed that both KDM3A and G9A could be pulled down by MYC‐SOX9 but not by MYC peptide as a negative control (Figure 5D,E). These results suggest that KDM3A and G9A could directly interact with SOX9 both in vitro and in vivo.

Based on the demonstrated interactions between KDM3A, G9A, and SOX9, we postulated that SOX9 could potentially be demethylated by KDM3A and methylated by G9A. To substantiate this hypothesis, we examined the alteration of the endogenous lysine methylation level of SOX9 after manipulating KDM3A or G9A expression. KDM3A was depleted by transfection with its specific siRNA, and then cell lysates were immunoprecipitated using the antibody against methylated lysine or IgG as a negative control. Immunoblotting with SOX9 antibody showed that lysine methylation of SOX9 increased significantly after KDM3A depletion (Figure 5F). The administration of MG132, a potent inhibitor of proteasomal degradation, further solidified this point (Figure 5G). Concordantly, stable knockdown of KDM3A using lentiviral shRNA also led to enhanced lysine methylation of SOX9 (Figure 5H). Conversely, overexpression of KDM3A caused an apparent reduction in SOX9 methylation (Figure 5H). These data support the notion that SOX9 is demethylated by KDM3A. As for G9A, immunoprecipitation assays indicated that siRNA‐mediated silencing of G9A caused a lower level of SOX9 lysine methylation in DPSCs (Figure 5I), implying that G9A could positively regulate the methylation level of SOX9.

To determine whether G9A could directly methylate SOX9, we performed an in vitro methylation assay by incubating G9A with an increased dose of SOX9, together with the methyl group donor S‐adenosyl‐l‐methionine (SAM). Using western blotting with a lysine methylation antibody, we noticed a remarkable methylation signal detected at the position of MYC‐SOX9 corresponding to its molecular weight, which could only be observed in the presence of G9A (Figure 5J), indicating that SOX9 could be methylated by G9A. After the methylation reaction, the SOX9 protein was further subjected to mass spectrometry analysis to detect the precise methylation site. Results indicated that K68 of SOX9 was mono‐methylated by G9A, which is well‐conserved among multiple species (Figure 5K,L). To determine whether KDM3A was able to demethylate SOX9 at this site, a dot blot assay was performed by incubating purified KDM3A protein with synthesized SOX9 peptide with mono‐methylated K68. The results revealed a significantly decreased methylation level of SOX9 peptide (Figure 5M), demonstrating that SOX9 could be demethylated by KDM3A at K68.

To examine whether SOX9 was modulated by KDM3A and G9A at K68 in vivo, a plasmid encoding mutant MYC‐SOX9 with K68 mutated into R was constructed. HEK293T cells were infected with lentivirus expressing shRNA of KDM3A or scrambled control (shNC) and then transfected with wild‐type or mutant SOX9 plasmid. Immunoprecipitation followed by western blotting indicated that knockdown of KDM3A led to upregulated methylation of SOX9 in cells transfected with wild‐type SOX9, whereas this promotion effect was completely undetectable in cells transfected with mutant SOX9 (Figure 5N). Likewise, compared to the strengthened methylation induced by G9A overexpression in cells transfected with wild‐type SOX9, the methylation level remained unchanged upon G9A overexpression in the case of mutant SOX9 (Figure 5O). These results indicate that SOX9 is methylated by G9A at K68, and demethylated by KDM3A at the same site both in vitro and in vivo.

### 2.6 Lysine Methylation Status Controlled by KDM3A and G9A Regulates Ubiquitination and Degradation of SOX9

**Figure 6:**
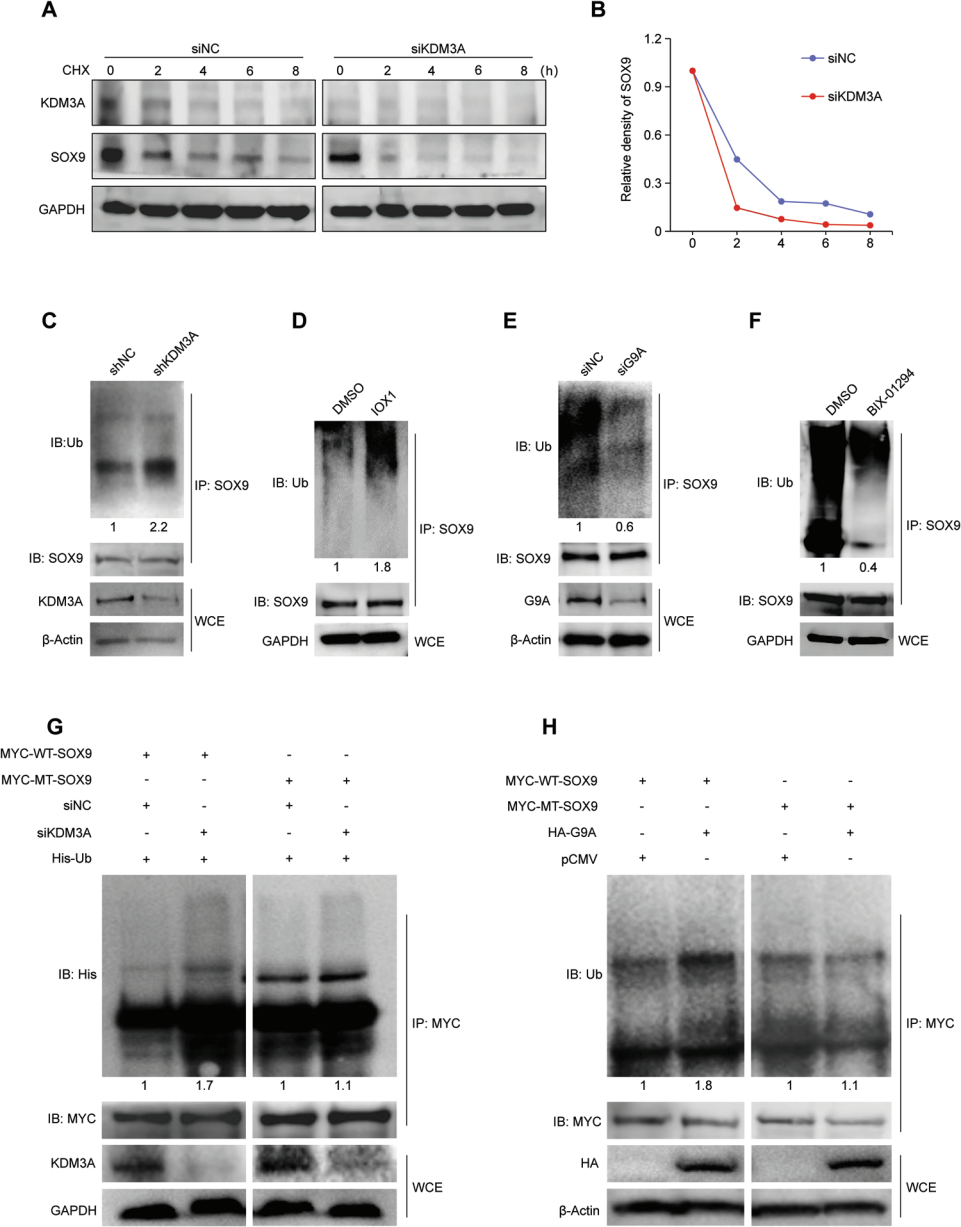
KDM3A and G9A affect poly‐ubiquitination and degradation of SOX9. A) DPSCs transfected with indicated siRNAs were treated with cycloheximide (CHX, 40 µg mL^−1^) for indicated times, and harvested for western blotting. B) The relative density of SOX9 protein bands shown in (A) was analyzed using Image J software. C) Western blotting showing extensive ubiquitination in KDM3A‐depletion DPSCs treated with MG132 (20 µm) for 5 h. D) DPSCs were exposed to IOX1 (0.1 µm), or DMSO (1.1 mg mL^−1^), and these cells were further treated with MG132 (20 µm) for 5 h. Protein extracts from these cells were subjected to an immunoprecipitation experiment with SOX9 antibodies used for immunoprecipitation and anti‐ubiquitin (Ub) antibodies used for immunoblotting. E) Western blotting showing decreased ubiquitination in G9A‐depletion DPSCs treated with MG132 (20 µm) for 5 h. F) Western blotting showing decreased ubiquitination in DPSCs treated with BIX‐01294 (1 µm) for 24 h and MG132 (20 µm) for 5 h. G,H) Post‐transfection with indicated plasmids or siRNA, HEK293T cells were further treated with MG132 (20 µm) for 5 h. The cells were then collected for immunoprecipitation with anti‐MYC antibody to enrich SOX9 proteins. Ubiquitination of SOX9 was then examined by immunoblotting analysis using anti‐His and anti‐Ub antibodies. The relative densities of proteins indicated below the blots were first normalized to that of reference proteins and then calculated as a ratio relative to the value in control cells. WT: wild type; MT: mutant type.

The crosstalk between distinct post‐translational modifications led us to hypothesize that KDM3A‐ and G9A‐mediated methylation may affect the ubiquitination of SOX9. To test whether SOX9 stability was regulated by KDM3A, KDM3A‐depleted DPSCs were treated with cycloheximide (CHX) to inhibit protein synthesis, followed by chasing the remaining SOX9. Western blotting showed that KDM3A silencing substantially reduced SOX9 protein stability, as revealed by the shorter half‐life and accelerated degradation of SOX9 compared with that of control cells (**Figure** **6**A,B). Moreover, treatment with the proteasome inhibitor MG132 resulted in SOX9 accumulation, indicating that SOX9 could be degraded via the ubiquitin‐proteasome pathway in DPSCs (Figure S10, Supporting Information).

To evaluate the effect of KDM3A on the ubiquitination of SOX9, we performed an in vivo ubiquitination assay in DPSCs after KDM3A knockdown using lentiviral shRNA. Western blotting showed increased ubiquitination upon KDM3A depletion (Figure 6C). To further confirm this point, we adopted 5‐carboxy‐8‐hydroxyquinoline (IOX1), an inhibitor of Jumonji C domain histone lysine demethylases (JmjC‐KDMs) including KDM3A. Treatment with IXO1 led to comparable enhanced ubiquitination (Figure 6D). These results imply that KDM3A deficiency promotes the ubiquitination of SOX9. Reciprocally, G9A knockdown significantly suppressed the ubiquitination of SOX9 (Figure 6E). Moreover, BIX‐01294 resulted in an obvious decrease in SOX9 ubiquitination (Figure 6F), suggesting that KDM3A contributes to SOX9 stabilization, whereas G9A promotes its degradation.

To clarify whether KDM3A‐ and G9A‐tuned lysine methylation were involved in ubiquitination regulation, HEK293T cells were transfected with siRNA against KDM3A or the control siNC, in conjugation with the plasmid encoding MYC‐tagged wild‐type SOX9 or mutated SOX9 (K68R). Results demonstrated that KDM3A knockdown significantly stimulated the incorporation of ubiquitin into wild‐type SOX9, whereas no obvious changes were observed in the ubiquitination of mutated SOX9 upon KDM3A depletion (Figure 6G). Similarly, ectopic expression of G9A also led to enhanced ubiquitination of wild‐type SOX9 but had no effect on that of SOX9 with the K68R mutation (Figure 6H). In summary, these data suggest that KDM3A‐mediated demethylation contributes to SOX9 stability maintenance by inhibiting SOX9 ubiquitination, whereas G9A inversely controls this process by methylating SOX9.

### 2.7 KDM3A and G9A Regulate the Chondrogenic Differentiation Capacity of DPSCs in a Rat Knee Cartilage Defect Repair Model

**Figure 7:**
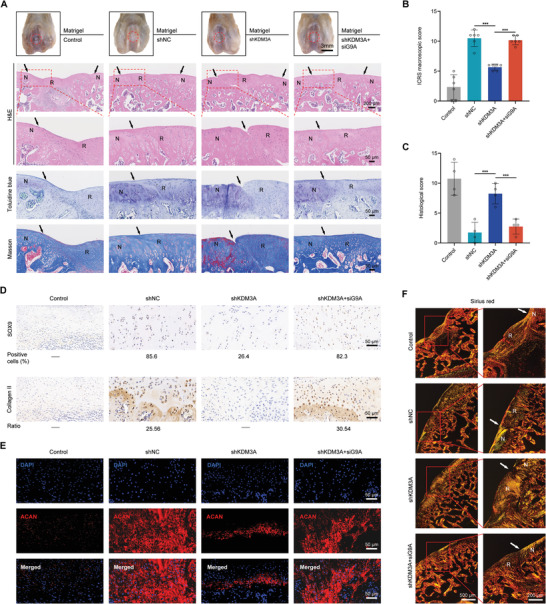
KDM3A and G9A regulate the chondrogenic differentiation capacity of DPSCs in vivo. A) Representative macroscopic analysis and histological staining (H&E, toluidine blue, and Masson staining) of repaired tissues at 8 weeks post‐operation (*n* = 6). Scale bars: 3 mm, 100 µm, and 50 µm. B) ICRS score for the macroscopic assessment. Data are presented as the mean ± SD (*n* = 6). C) Histological scores for histological evaluation after 8 weeks of cartilage repair. Data are presented as the mean ± SD (*n* = 4). D) SOX9 and collagen II immunohistochemical staining after 8 weeks of cartilage repair. The percentage of immunopositive cells or the relative chromogen intensity is indicated. Scale bars: 50 µm. E) Aggrecan (ACAN) immunofluorescence staining after 8 weeks of cartilage repair. F) Sirius red staining showing collagen formation of repair tissues. Red circles show the defect area. Solid arrows indicate the repair interface. H&E: hematoxylin‐eosin. N: normal cartilage; R: repaired cartilage. Each bar represents mean ± SD; ***p < 0.001.

We next evaluated the effect of KDM3A and G9A inhibition on the stem cell‐based restoration of hyaline cartilage. A full‐thickness osteochondral lesion was filled with only Matrigel (control), or Matrigel containing DPSCs transduced with lentiviruses (shNC, shKDM3A, shKDM3A+siG9A). After 8 weeks, cartilage defects in the control group without DPSCs injection remained obvious. But in the shNC group, the defects were partially repaired as revealed by the new tissues formed at the lesion site, indicating an effective DPSC‐mediated cartilage regeneration. The repair effect was largely impaired upon KDM3A depletion, as seen from the obvious defects and uneven surface analogous to that in the control group. When G9A was knocked down together with KDM3A, the degree of repair was significantly better than that in the shKDM3A group, as the boundary between the newly formed tissues and the surrounding normal cartilage was vague and difficult to distinguish (**Figure** **7**A and Figure S11, Supporting Information), suggesting G9A knockdown could rescue the reduced cartilage repair capacity of DPSCs caused by KDM3A deletion. Visually, the ICRS macroscopic score of the shKDM3A+siG9A group was nearly twice that of the shKDM3A group (Figure 7B).

Histomorphological staining was used to determine the properties of repaired articular cartilage tissue. Consistent with the results of the macroscopic evaluation, hematoxylin‐eosin (H&E) staining showed that the repaired area was still not well integrated with normal cartilage, and partial collapse was detected in the shKDM3A group (Figure 7A). Reciprocally, the repaired cartilage area was well integrated with normal cartilage, and chondrocytes were clearly aligned in shNC and shKDM3A+siG9A groups. Lesions in the shKDM3A group failed to fully recover the organization of hyaline cartilage and exhibited features of fibrocartilage with little cartilage matrix and a large number of disorganized collagen fiber bundles (Figure 7A). In contrast, the repaired area in the shKDM3A+siG9A group was similar to that of the normal cartilage in both matrix content and fibrous arrangement (Figure 7A). Consistent with the staining results, histological scores were lower in the shKDM3A+siG9A group than in the control group (Figure 7C). The regenerative tissues derived from the shKDM3A+siG9A group exhibited robust expression of cartilage‐specific matrix proteins and SOX9 compared with the fibrocartilage originating from the shKDM3A group (Figure 7D,E and Figure S12, Supporting Information). Moreover, the total collagen content of the repaired tissues was verified by sirius red staining (Figure 7F). Birefringent collagen fibers were observed in the repaired area of the shKDM3A+siG9A group, and these molecules were tightly and regularly arranged. Conversely, flocculent fibrous tissue was present in the shKDM3A group, and these fibers were sprawled and had gaps with the normal tissue. Micro‐CT analysis was performed to further verify the effect of KDM3A and G9A on the tissue repair capacity of DPSCs in vivo. Reconstruction images showed that the repaired tissue in the shKDM3A+siG9A group was much greater than that in the shKDM3A group at 8 weeks after the operation (Figure S13A, Supporting Information). The two important indices bone volume fraction (BV/TV) and bone mineral density (BMD), also confirmed this finding (Figure S13B,C, Supporting Information). These results indicate that KDM3A promotes articular cartilage in situ repair, whereas G9A has the opposite effect.

### 2.8 BIX‐01294 Stimulates Chondrogenesis of DPSCs

**Figure 8:**
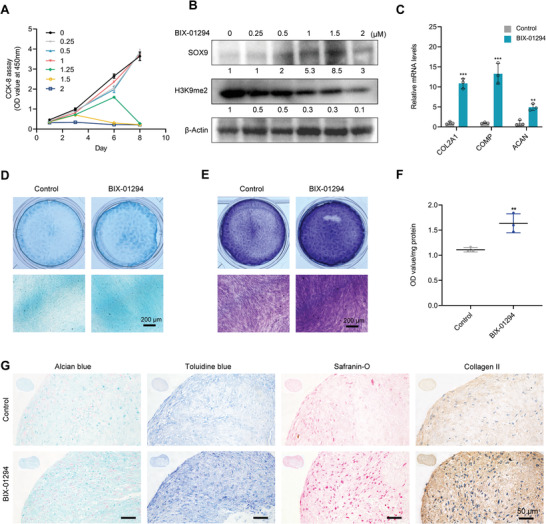
BIX‐01294 promotes chondrogenic differentiation of DPSCs. A) In vitro cytotoxicity of BIX‐01294. B) Western blotting showing total protein level of H3K9me2 and SOX9 in DPSCs treated with stepwise increasing concentrations of BIX‐01294. C) RT‐qPCR indicating enhanced mRNA level of cartilage‐signature markers in DPSCs after BIX‐01294 (1 µm) treatment. D,E) Alcian blue (D) and toluidine blue (E) showing augmented cartilage matrix after 2 weeks of chondrogenic induction with BIX‐01294 (1 µm). Scale Bars: 200 µm. F) Quantification of proteoglycans synthesis obtained from (E). G) Histomorphological staining showing elevated proteoglycans and collage II after BIX‐01294 (1 µm) treatment in DPSC pellets. Scale Bars: 50 µm. The relative densities of proteins indicated below the blots were first normalized to that of internal reference proteins and then calculated as a ratio relative to the value in control cells. Each bar represents mean ± SD; *n* = 3 per group; **p < 0.01, ***p < 0.001.

As mentioned above, the SOX9 protein level was also boosted by the pharmacological inhibition of G9A activity through treatment with BIX‐01294 (Figure 4E). Therefore, it was likely that the G9A inhibitor BIX‐01294 could stimulate the chondrogenic differentiation of DPSCs. To verify this, CCK‐8 assay and western blotting were first conducted to determine the appropriate concentration of BIX‐01294 with both validity and biosafety. As shown in **Figure** **8**A, the proliferation of DPSCs was not affected by BIX‐01294 treatment at concentrations lower than 1 µm, but striking cytotoxicity was detected at concentrations higher than 1 µm. Meanwhile, stepwise increasing the dosage of BIX‐01294 effectively attenuated the expression of histone H3K9me2, accompanied by enhanced SOX9 protein level (Figure 8B). Eventually, 1 µm BIX‐01294 was used to assess its effect on the chondrogenic differentiation of DPSCs.

Cultured under chondrogenic induction conditions, we found that DPSCs treated with BIX‐01294 showed enhanced chondrogenic differentiation capacity, as indicated by the augmented expression levels of cartilage‐signature markers (Figure 8C) and markedly increased proteoglycans (Figure 8D‐F). In addition, BIX‐01294 effectively induced chondrogenic nodule formation in the micro‐mass culture of DPSCs, as revealed by enhanced binding to alcian blue, toluidine blue, and safranin‐O to sulfated glycosaminoglycans (Figure 8G). Immunohistochemical analysis showed that BIX‐01294 treatment led to an elevated expression of collagen II (Figure 8G). Taken together, BIX‐01294 exhibits an excellent ability to promote DPSCs chondrogenesis.

## 3 Discussion

Various shreds of evidence support the notion that epigenetic mechanisms regulate self‐renewal and direct the terminal fate of stem cells.^[^ ^49^, ^50^ ^]^ However, incomplete epigenetic regulatory networks and unclear key regulatory factors limit the promotion of cartilage tissue engineering into clinical applications.^[^ ^51^ ^]^ In this study, for the first time, we used transcriptomics to reveal genome‐wide alterations during the chondrogenic differentiation of DPSCs. Through a series of epigenomic and molecular biology approaches, we delineated KDM3A and G9A as antagonistic regulators of DPSCs chondrogenesis. To date, no one has reported before that KDM3A and G9A are capable of post‐translationally modifying SOX9, altering SOX9 ubiquitination by regulating lysine methylation at K68 residue and ultimately affecting its protein stability. The findings presented here revealed that the lysine methylation of SOX9 mediated by KDM3A and G9A is required to maintain the chondrogenic differentiation capacity of DPSCs in vitro and in vivo.

DPSCs have demonstrated their chondrogenic and reparative abilities through in vitro and in vivo studies,^[^ ^16^, ^17^, ^18^, ^52^ ^]^ but the transcriptomic alterations and associated epigenetic regulatory networks during their chondrogenic differentiation remain unexplored. In our study, transcriptomics revealed significant changes in ECM genes during chondrogenic differentiation, which is consistent with previous studies.^[^ ^53^, ^54^ ^]^ It is widely believed that the ECM regulates stem cell differentiation and that the derived decellular matrix also contributes to MSCs chondrogenesis. GO analysis also revealed that the shared downregulated genes were enriched in the regulation of immune response. It is well established that immunomodulatory properties are significant properties of DPSCs, which make them able to address a diverse set of autoimmune and inflammatory diseases.^[^ ^14^, ^55^ ^]^ Recently, a study demonstrated that Fas/FasL pathway is not only involved in determining the immunomodulatory properties but also in supporting the chondrogenic differentiation of DPSCs.^[^ ^13^ ^]^ Further transcriptomic analysis and in‐depth exploration of immune‐related genes can provide a new direction for the study of immunomodulatory characteristics of DPSCs in the future. At the same time, KEGG pathway analysis revealed significant enrichment of PI3K‐Akt, MAPK, and Wnt signaling pathways in the overlapped DEGs, indicating that the signaling pathways orchestrating chondrogenesis in DPSCs are highly comparable to those in other types of MSCs, such as BMSCs. In addition to KDM3A, we noticed an altered expression of other epigenetic regulators during chondrogenic differentiation including KDM7A, HDAC9, HDAC10, NCOA3, CHD5, CHD7, and LSH. It is plausible to speculate that these genes potentially play an important role in modulating epigenetic alterations in DPSCs, providing directions for future studies on the regulatory network of stem cell chondrogenesis.

Although studies on protein methylation have thus far focused on histone modifications, the essential role of non‐histone methylation in cellular pathways and protein activity has recently attracted strong interest from researchers and the public.^[^ ^56^, ^57^ ^]^ Study has shown that KDM3A inhibits the tumor suppressor p53 transcriptional activity by demethylating K372, thus impeding p53‐mediated transcription and pro‐apoptotic functions.^[^ ^32^ ^]^ In contrast, the C‐terminal lysine residue of p53 is methylated at K373 by G9A and GLP.^[^ ^58^ ^]^ Moreover, it has been reported that KDM3A cooperates with G9A to regulate the reactivation of octamer–binding transcription factor 4 (OCT4) during embryonic stem cell‐induced reprogramming.^[^ ^47^ ^]^ To date, there are no studies on the role of KDM3A and G9A in non‐histone modifications during stem cell differentiation. In this study, we revealed the previously unrecognized role of KDM3A and G9A in modifying non‐histone SOX9. KDM3A demethylates non‐histone SOX9 to enhance its stability, whereas G9A methylates SOX9 to promote its degradation.

Crosstalk between lysine methylation and other post‐translational modifications has important regulatory implications in gene expression, heterochromatin, genome stability, cancer, etc.^[^ ^59^ ^]^ Generally, the crosstalk between lysine methylation and ubiquitination regulates biological functions by affecting protein stability.^[^ ^57^ ^]^ On one hand, lysine methylation is capable of promoting protein stability by blocking potential sites of ubiquitination.^[^ ^60^ ^]^ For example, SET9‐mediated lysine methylation of p53 at K372 promotes its protein stability by interfering with ubiquitination.^[^ ^61^ ^]^ Recently, it was shown that EZH2 mediates lysine 295 methylation of forkhead box A1 (FOXA1), an essential prostatic developmental regulator. WD40 repeat protein BUB3 recognizes this methylation and subsequently recruits ubiquitin‐specific protease 7 to remove ubiquitination and enhance FOXA1 protein stability.^[^ ^62^ ^]^ On the other hand, lysine methylation can also play the opposite role as a protein degradation signal by recruiting the ubiquitin ligase machinery, directly or indirectly, through adaptor proteins.^[^ ^63^ ^]^ For example, lysine methylation of FOXO1 by G9A reduces its protein stability by increasing the interaction between FOXO1 and a specific E3 ligase, SKP2, resulting in enhanced FOXO1 ubiquitination.^[^ ^35^ ^]^ Similarly, our data strongly suggested that KDM3A‐mediated lysine demethylation of SOX9 maintains its protein stability by decreasing SOX9 ubiquitination, while G9A conversely controls this process antagonizing KDM3A.

Post‐translational modifications of SOX9 are closely related to its activity or degradation, leading to functional abnormalities in MSCs or chondrocyte precursor cells in chondrogenic differentiation. Currently, identified post‐translational modifications of SOX9 include phosphorylation, arginine methylation, SUMOylation, ubiquitination, acetylation, and deacetylation, which affect its trans‐activation activity, DNA‐binding affinity, and stability.^[^ ^27^, ^64^, ^65^ ^]^ We revealed a previously unrecognized role of lysine methylation in the regulation of SOX9 degradation. In agreement with the proteasome‐dependent degradation of SOX9, we showed that KDM3A and G9A interact with SOX9 to regulate its ubiquitination in a methylase activity‐dependent manner. However, the mechanism by which K68 methylation promotes SOX9 ubiquitination remains unclear. In search of current knowledge on the molecular mechanisms governing SOX9 ubiquitination, we found that UBE3A acts as a ubiquitin ligase for SOX9 in chondrocytes.^[^ ^26^ ^]^ In addition, SOX9 has been shown to be targeted for proteasomal degradation by the E3 ligase FBW7 in response to DNA damage.^[^ ^66^ ^]^ SUMO–specific protease 2 and Kelch–like ECH–associated protein 1 promote the ubiquitination of SOX9 in cancer cells.^[^ ^67^, ^68^ ^]^ On the contrary, SOX9 ubiquitination is negatively controlled by heat shock protein 60 and DDRGK domain containing 1 in chondrocytes.^[^ ^65^, ^69^ ^]^ Based on these findings, we speculate that SOX9 methylation at K68 might lead to changed interaction between SOX9 and the abovementioned ubiquitination regulators or some unknown factors that await further investigation. Thus, we will examine these possibilities and attempt to unravel the key factors mediating K68 methylation‐dependent SOX9 ubiquitination in our future work.

BIX‐01294 as a specific inhibitor of G9A has been studied extensively. BIX‐01294 has been discovered as a potent anticancer drug via G9A inhibition, leading to cell death in G9A‐overexpressing bladder, lung, and breast cancer cells.^[^ ^70^, ^71^, ^72^ ^]^ The underlying mechanism may be that BIX‐01294 induces ROS‐mediated autophagy and cell death in cancer cells.^[^ ^73^ ^]^ Furthermore, BIX‐01294 was previously used as a replacement for OCT3/4 to generate induced pluripotent stem cells.^[^ ^74^ ^]^ Culmes et al. investigated the role of BIX‐01294 in the differentiation of adipose‐derived MSCs.^[^ ^75^ ^]^ Their results suggested that BIX‐01294 can reduce global DNA methylation and unfolding chromatin by altering histone methylation, thereby promoting endothelial differentiation of adipose‐derived MSCs. In this study, BIX‐01294 was employed to reverse the effect of KDM3A depletion and to rescue the decreased expression of SOX9. Further studies showed that BIX‐01294 is capable of promoting chondrogenic differentiation of DPSCs by inhibiting SOX9 degradation. Crucially, our data provide a preliminary insight into the possible use of BIX‐01294 as a chondrogenic differentiation promoter and G9A as a potential therapeutic target for cartilage defect treatment.

Intriguingly, our study showed that SOX9 protein was accumulated by lentiviral enforced expression of KDM3A in hBMSCs (Figure S7, Supporting Information), indicating the probability that modulation of SOX9 by KDM3A not only occurred in DPSCs but also broadly existed in other types of MSCs. Hence, in the future, it can be anticipated that the development of small‐molecule activators specific to KDM3A could be used in MSC‐based chondrocyte regeneration to gain improved therapeutic effects. Moreover, through transcriptomic analysis of the time‐course chondrogenic differentiation of DPSCs, we discovered a series of TFs that were significantly expressed upon chondrogenic induction. It is possible that KDM3A might have a potential regulatory role in these TFs, which are important for DPSCs chondrogenesis and need further investigation in the future. Overall, our study identified KDM3A and G9A as antagonistic regulators of the chondrogenic differentiation of DPSCs and clarified the lysine methylation of SOX9 mediated by KDM3A and G9A, which further affected the ubiquitination and degradation of SOX9. These results expand our understanding of epigenetic regulation of MSC chondrogenesis and provide novel molecular targets for the amelioration of MSCs application in cartilage repair.

## 4 Experimental Section

A detailed Experimental Section can be found in the Supporting Information.

## Conflict of Interest

The authors declare no conflict of interest.

## Author Contributions

B.H. and Q.L. conceived and oversaw the project. B.H., Q.S., and Q.L. designed experiments. Q.S., Z.Z., and R.B. performed experiments. Q.S., J.D., Z.Z., T.X., and Y.Z. analyzed data. Q.S. and Q.L. wrote the manuscript. Q.L. analyzed the RNA‐Seq data. All authors contributed to reading and editing the manuscript.

## Supporting information

Supporting Information

## Data Availability

The data that support the findings of this study are available from the corresponding author upon reasonable request.
