# Supplementary material for: Lysine 68 Methylation‐Dependent SOX9 Stability Control Modulates Chondrogenic Differentiation in Dental Pulp Stem Cells
Source: Adv Sci (Weinh). 2023 Jun 29;10(24):2206757. doi: 10.1002/advs.202206757 (PMC10460901; doi:10.1002/advs.202206757)
Supplement: Supplementary file 1 — Supporting Information [file ADVS-10-2206757-s001.pdf]

ADVANCED SCIENCE
Open Access

Supporting Information

for *Adv. Sci.*, DOI 10.1002/advs.202206757

Lysine 68 Methylation-Dependent SOX9 Stability Control Modulates Chondrogenic Differentiation in Dental Pulp Stem Cells

Qiannan Sun, Zimeng Zhuang, Rushui Bai, Jie Deng, Tianyi Xin, Yunfan Zhang, Qian Li and Bing Han**

Supporting Information

Lysine 68 Methylation-dependent SOX9 Stability Control Modulates Chondrogenic Differentiation in Dental Pulp Stem Cells

Qiannan Sun, Zimeng Zhuang, Rushui Bai, Jie Deng, Tianyi Xin, Yunfan Zhang, Qian Li, Bing Han**

Department of Orthodontics, Peking University School and Hospital of Stomatology, Beijing, 100081, China.

E-mail: kqbinghan@bjmu.edu.cn; qianli@bjmu.edu.cn

Table of contents

Experimental Section 3

Cell Culture and Treatment 3

Flow cytometry 3

RNA-sequencing 3

Small Interfering RNAs, Plasmid Transfection, and Viral Infection 4

Quantitative Reverse-transcription Polymerase Chain Reaction (RT-qPCR) 4

Co-immunoprecipitation (Co-IP) and Western Blotting 4

Indirect Immunofluoresence (IF) 5

In Vitro Chondrogenic Differentiation Capacity 6

Cycloheximide (CHX) Chase Assay 6

In Vitro Methylation and Dot-blot Assays 6

In Vivo Ubiquitination Assay 7

Evaluation of Cartilage Regeneration in a Rat Knee Cartilage Defect Model 7

Histological Analysis 8

Statistical Analysis 9

Table S1, Figures S1-S13 9

Reference 16

Experimental Section

Cell Culture and Treatment

Human dental pulp stem cells (DPSCs) were obtained from dental stem cell bank (Beijing Taisheng Biological Technology Co., LTD). DPSCs were cultured in α-minimum essential medium (α-MEM, Gibco, USA) with 1% penicillin streptomycin (Gibco) and 10% fetal bovine serum (FBS, Procell, China) in a 5% CO_2 incubator at 37 °C. All the experiments were performed using DPSCs between P4 and P8, to ensure that the cells were functionally stable. HEK293T cells were donated by the Department of Biochemistry and Molecular Biology, Peking University Health Science Center. They were cultured in high glucose Dulbecco's modified eagle's medium (DMEM, Gibco) supplemented with 1% penicillin streptomycin and 10% FBS in a 5% CO_2 incubator at 37 °C. DPSCs and HEK293T cells transfected with siRNA, plasmid and lentivirus were cultured in basic medium containing only 10 % FBS. MG132 (S2619) was purchased from Selleck. Blasticidin S (HY-103401), BIX-01294 (HY10587), and IOX1 (HY12304) were purchased from MedChemExpress. Cycloheximide (CHX, C104450) was purchased from Sigma. RNase A (ST576) was purchased from Beyotime. The concentrations of drugs used are indicated in the relevant figure legends.

Flow cytometry

The immunophenotype of obtained DPSCs at 4th passage (P4) were evaluated by flow cytometry. In short, DPSCs were isolated by incubation with trypsin (Gibco, USA) and resuspended in FACS staining buffer (Transgen, China). The antibodies used for FACS analysis were mouse anti-human CD45_PerCP-Cy5.5, CD73_FITC, CD90_APC, CD105_PE, and the corresponding mouse IgG1 subtype bound to fluorescein isothiocyanate or phycoerythrin. The cells were stained at room temperature for 30 min and then washed with staining buffer. Calibur (BD, USA) was used for detection. The data were analyzed using Flow Jo.

RNA-sequencing

After culture in chondrogenic induction media (Thermo Fisher Scientific, USA) for 0, 3, 7 and 14 days, 1×10^6 DPSCs were resuspended in TRIzol ((Invitrogen), and stored at -80 °C. Three RNA samples from each time point were used for the RNA sequencing. RNA extraction, library preparation and sequencing were conducted by the Beijing Genome Institute (BGI).

Small Interfering RNAs, Plasmid Transfection, and Viral Infection

DPSCs and HEK293T cells were transfected with Silencer® Select Small interfering RNA (siRNA, GenePharma, China) targeting KDM3A (siKDM3A), G9A (siG9A) and scrambled control (siNC) using Lipofectamine™ RNAiMAX (Thermo Fisher Scientific). Cells were harvested 48 h after transfection and used for western blotting analysis of knockdown efficiency and total RNA extraction for gene expression analysis.

Cells that had reached 70 % to 80 % confluency were transiently transfected with plasmids using Lipofectamine 3000 (Thermo Fisher Scientific) as per the manufacturer's instructions.

Short hairpin RNA (shRNA) targeting KDM3A (shKDM3A), shRNA-scrambled control (shNC), adenoviruses expressing KDM3A, G9A and the control vector were constructed by GenePharma. The cells were cultured until confluence reached approximately 30–50 %, and transfection was conducted. They were divided into virus transfection and empty virus groups. After 12 h, the fresh medium was replaced and cell growth was observed under a microscope. Green fluorescence was observed under an inverted fluorescence microscope 3 days after transfection. When the confluence reached 80–90 %, the cells were stored for passaging via 1:3 pass. Blasticidin S (10 μg/mL) was added to the media the next day, and the screening medium was replaced every 2 days. When all cells in the untransfected group died, the Blasticidin S concentration was reduced to 1 μg/mL and the selection pressure was maintained.

Quantitative Reverse-transcription Polymerase Chain Reaction (RT-qPCR)

RNAs were extracted from the cultured cells using TRIzol. Full-length cDNA was synthesized using a cDNA synthesis kit (Transgen, China). qPCR was performed according to the manufacturer's instructions (Transgen, China), using three independent RNA preparations as biological replicates. Human GAPDH gene transcripts were used as an internal control. The relevant primer sequences are given in Table S1. All the primers used for RT-qPCR are available upon request.

Co-immunoprecipitation (Co-IP) and Western Blotting

For the Co-IP, cells were collected and lysed on ice with lysis buffer containing a protease inhibitor cocktail (Transgen, China). The lysates were pre-cleared by incubation with protein A/G agarose beads (Transgen, China). The protein complex was precipitated using a specific antibody together with protein A/G beads followed by extensive washing. Western blotting was conducted to analyze the resulting materials.

For western blotting, cells were harvested, and lysed by vortexing in RIPA lysis buffer (Beyotime, China) containing a protease inhibitor cocktail. Lysate protein was quantified using the BCA method (Thermo Fisher Scientific). Equal amounts of proteins were used for electrophoresis on 10 % SDS-PAGE gels and transferred to PVDF membranes (Millipore) for 2 h at 200 mA. The membrane was blocked with 5% skim milk prepared in TBST and incubated overnight with primary antibodies at 4℃. The membranes were then incubated with HRP-conjugated secondary antibodies for 1 h at room temperature. Anti-methylated lysine (ab23366, diluted 1:1000) and anti-SOX9 (ab185230, diluted 1:500) were bought from Abcam, UK; anti-MYC (HT101-01, diluted 1:2000), anti-HA (HT301-01, diluted 1:1000) and anti-His (HT501-01, diluted 1:1000) from Transgen, China; anti-polyubiquitin (20326, diluted 1:1000), anti-IgG (2729, diluted 1:2000) and anti-FLAG (14793, died 1:2000) from Cell Signaling Technology, USA; anti-G9A (29303-1-AP, diluted 1:500), anti-aggrecan (13880-1-AP, diluted 1:1000), anti-collagen type II (28459-1-AP, diluted 1:1000) and anti-KDM3A (12835-1-AP, diluted 1:500) from Proteintech, USA; anti-β-Actin (sc-47778, diluted 1:2000) from Santa Cruz, USA; anti-GAPDH (AC001, diluted 1:2000) and anti-α-Tubulin (AC007, diluted 1:2000) were purchased from ABclonal, China; goat anti-mouse IgG/HRP (SE131, diluted 1:10000) and goat anti-rabbit IgG/HRP (SE134, diluted 1:10000) were purchased from Solarbio, China.

Indirect Immunofluoresence (IF)

For monolayered cells, DPSCs were fixed using 4 % paraformaldehyde, permeabilized with 0.2 % Triton X-100 (Invitrogen, USA) and blocked with 3 % PBS-diluted Albumin Bovine V (BSA) for 45 min at room temperature. Subsequently, the cells were incubated with primary antibodies followed by incubation with FITC/RBITC–conjugated secondary antibodies. Cells were mounted in a glycerol/PBS solution containing 4′,6-diamidino-2-phenylindole (DAPI, Solarbio, China). Confocal images were obtained using a Zeiss LSM confocal laser-scanning microscope.

For three-dimensional cells, 5 × 10^5 cells were evenly mixed with 400 mL Matrigel (Corning, USA) and seeded in 24-well plate. After 30 min of incubation, the appropriate amount of complete medium was added. After culture with Matrigel for 48 h, cells were fixed in 4 % paraformaldehyde. The next procedure was the same as described above for monolayer cells.

In Vitro Chondrogenic Differentiation Capacity

For monolayer cultured cells, DPSCs were cultured in complete medium until they reached sub-confluence. The medium was then replaced by stem cell chondrogenic differentiation media and the medium were changed every 3 days. Then cells were rinsed with PBS and fixed in 4% paraformaldehyde for 30 min. After fixation, cells were rinsed with PBS and stained with 1% alcian blue or toluidine blue for 30 min. Cells were rinsed with PBS to neutralize the acidity; after adequate washing, they were visualized under a light microscope, and images were captured for analysis. To quantify proteoglycan synthesis, alcian blue or toluidine blue was extracted using 4 M guanidine-HCl overnight at 4°C. Absorbance values were read at 600 nm after temperature equilibration. The final OD value in each group was normalized with the total protein concentrations prepared using a duplicate plate.[1]

For culture of cell pellets, 3 × 10^5 transfected DPSCs were centrifuged at 250 *g* for 5 min in a 15 mL conical tube to obtain pellets. The DPSC pellets were cultured in stem cell chondrogenic differentiation media for up to 14 days. The medium was changed every 2 days. After 14 days of differentiation induction, the cell pellets were sectioned into 5 μm frozen slices and stained with alcian blue, safranin O, toluidine blue and sirius red as per the manufacturer's standard protocols.

Cycloheximide (CHX) Chase Assay

For protein degradation analysis, the cells were treated with CHX (40 μg/mL), and aliquots of these cells were collected at specific time points. Western blotting was performed for evaluating the protein levels. Protein bands were analyzed using Image J.

In Vitro Methylation and Dot-blot Assays

The *in vitro* methylation assay was performed in accordance with the methodology specified in a previous study.[2] Recombinant GST-G9A (1 μg) was incubated with various amounts of SOX9 proteins or peptides in a methylation assay buffer (50 mM Tris-HCl, pH 8.0; 10 %

glycerol; 20 mM KCl; 5 mM $MgCl_2$; 1 mM dithiothreitol; 1 mM PMSF; 0.1 mM SAM) at 30 \°C for 4 h. The reactions were stopped by adding SDS-PAGE sample buffer, and the methylation status was analyzed using western blotting or mass spectrometry.

To test the ability of KDM3A to demethylate SOX9, a dot-blot assay was conducted. Recombinant GST-KDM3A (2 µg) was incubated with the SOX9 K68me peptide at 37 C for 4 h in the demethylation buffer [50 mM Tris-HCl, pH 8.5; 5% glycerol; 50 mM KCl; 5 mM $MgCl_2$; 1 mM PMSF; 0.5 % BSA]. First, the reaction in each group was blotted on a nylon membrane (Millipore; USA), blocked for 1.5 h in 5% skim milk, and washed with ice-cold PBS for 5 min. The membrane was then incubated overnight with the desired primary antibody at 4°C, after which the membrane was exposed to an appropriate HRP-conjugated secondary antibody for 1 h at room temperature, and the proteins were observed using an enhanced chemiluminescence reagent. K68me peptide sequence is: KESEEDK (me) FPVCIR.

In Vivo Ubiquitination Assay

In vivo ubiquitination assays were performed as described previously.[3] Briefly, HEK293T cells cultured in each 10 cm dish were transfected with different combinations of MYC-SOX9 (8 µg), His-ubiquitin (6 µg), and HA-G9A (6 µg), or siKDM3A. After 48 h of transfection, the cells were treated with MG132 (20 µM) for 5 h and then lysed with denaturing lysis buffer (50 mM Tris-HCl, pH 7.5; 0.5 mM EDTA; 1 mM DTT, 1 % SDS) by vortexing and boiling for 10 min. The supernatants were collected by centrifugation at 13,000 rpm for 10 min and diluted 10-fold with an NP-40 buffer (50 mM Tris, pH 7.5; 150 mM NaCl; 0.3% Nonidet P-40). Further, the lysates were immunoprecipitated with anti-SOX9, anti-His, anti-ubiquitin antibodies and protein G agarose beads. Western blotting was conducted to probe ubiquitylated proteins with anti-ubiquitin or anti-His antibodies.

Evaluation of Cartilage Regeneration in a Rat Knee Cartilage Defect Model

All animal experiments were carried out in compliance with the Animal Management Rules of the Ministry of Health of the People's Republic of China and conducted with the approval of Animal Ethics Committee of Peking University Health Science Center (LA2019313).

Sprague-Dawley (SD) rats (5-6 weeks old) were purchased from Charles River Company (Beijing, China). The patellae were laterally dislocated, full-thickness articular cartilage defect (2 mm in diameter and 1 mm in depth) was created at the middle of femoral trochlea using a 2 mm drill. After removing the cartilage and bone debris, boundaries around the drill

were trimmed using a surgical knife and rinsed to remove the debris. According to the type of shRNA and siRNA transfected with DPSCs, SD rats with cartilage defects were randomly divided into the following four groups: (1) Control; (2) shNC; (3) shKDM3A; (4) shKDM3A+siG9A (n = 6 per group). The treated/control DPSCs (2 × 10^6 cells/mL) in a low gelling temperature Matrigel (Corning, USA) were then transplanted into the cartilage defect. The Matrigel without cells was implanted as the control group. Following implantation, patellar retinaculum and overlying soft tissues were closed in layers. Animals were euthanized at 8 weeks post-implantation to assess the cartilage repair statuses. The cartilage defect area on the knee was observed and photographed. The degree of articular cartilage repair was scored by three experienced researchers who were blinded to the groups in accordance with the macroscopic evaluation guidelines recommended by the International Cartilage Repair Society (ICRS).[4]

The subchondral bones were excised and fixed in 4% neutral-buffered formaldehyde and then subjected to microcomputed tomography (μCT, SkyScan 1174) to detect bone formation. Indexes including bone mineral density (BMD) and bone volume to total volume (BV/TV) were quantitatively assessed.

Histological Analysis

The knee samples from the four groups were fixed in 4 % paraformaldehyde for 3 days, decalcified with 5% EDTA (pH 7.4) for 1 month, and continuously trimmed during the process. Then, the specimens were embedded in paraffin, sliced at a thickness of 5 μm and stained with hematoxylin-eosin (H&E), Masson's, toluidine blue, and Sirius red as per standard protocols. Histological sections stained with H&E and toluidine blue were used to assess the histological score of cartilage degeneration.[5]

Immunofluorescence (IF) and immunohistochemical (IHC) of the samples were performed as described previously.[6] In brief, after the sections were dewaxed and washed, endogenous peroxidase was removed with hydrogen peroxide, they were washed with PBS and blocked. The sections were incubated overnight at 4°C with anti-SOX9 (1:200, Abcam, UK), anti-Collagen Type II (1:200, Abcam, UK), and anti-aggrecan (1:200, Proteintech, USA) antibodies. Eventually, the sections were incubated with the secondary antibody for 1 h at room temperature. After dewaxing and sealing, the sections were observed and photographed under a microscope. Protein expression levels were semiquantitatively analyzed using ImageJ.

Statistical Analysis

Statistical analysis was performed with SPSS 24.0. All results are presented as the mean and standard deviation (mean ± SD) for n from 3 to 6. Data were assessed via independent 2-tailed Student's *t*-test or one-way analysis of variance. For all tests, $P < 0.05$ was considered significant.

Table S1, Figures S1-S13

Table S1. Sequences of primers used for qPCR.

Gene symbol	Forward primer (5′-3′)	Reverse primer (5′-3′)
KDM3A[a)]	TGAACATCGTGGCACAGTTG	AAGAAGCGGCAAAACACAGG
SOX9[b)]	TGCTGCTGGGAAACATTTGC	TTGGCTGCTGAAACATTCCC
ACAN[c)]	TGGACACCCCATGCAATTTG	TGCAGGGAACATCATTCCAC
COL2A1[d)]	ATGGCTGCACGAAACATGTC	ATGTCCATGGGTGCAATGTC
COMP[e)]	AAGGCCAACAAGCAGGTTTG	TGTTGATGCACACGGAGTTG
NANOG[f)]	CTCCATGAACATGCAACCTG	CTCGCTGATTAGGCTCCAAC
OCT4A[g)]	AGTGAGAGGCAACCTGGAGA	GTGAAGTGAGGGCTCCCATA
OCT4B[h)]	TATGGGAGCCCTCACTTCAC	CAAAAACCCTGGCACAAACT
SOX2[i)]	TCCACACTCACGCAAAAACC	AGTCCCCCAAAAAGAAGTCCAG
GADPH[j)]	AATTCCATGGCACCGTCAAG	ATCGCCCCACTTGATTTTGG

[a)]KDM3A, lysine demethylase 3A; [b)]SOX9, sex determining region Y-type high-mobility group box protein 9; [c)]ACAN, aggrecan; [d)]COL2A1, collagen alpha1(II) gene; [e)]COMP, cartilage oligomeric matrix protein; [f)]NANOG, Nanog homeobox; [g)]OCT4A, Octamer-binding transcription factor 4A; [h)]OCT4B, Octamer-binding transcription factor 4B; [i)]SOX2, SRY-box transcription factor 2; [j)]GAPDH, glyceraldehyde-3-phosphate dehydrogenase.

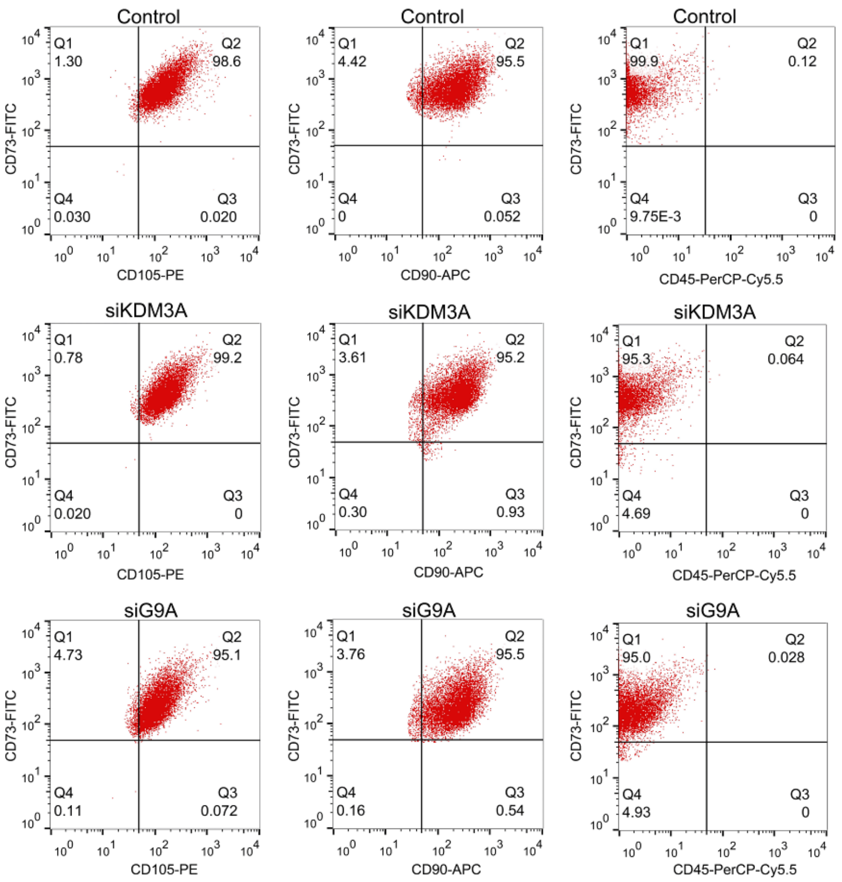

Figure S1. Flow cytometric analysis showing the expression of CD105, CD90, CD73 and CD45 in control, KDM3A-depleted and G9A-depleted DPSCs.

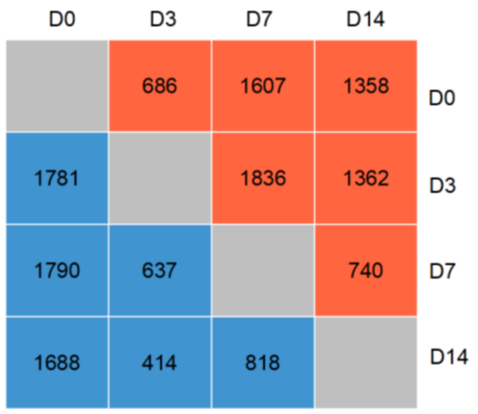

Figure S2. The number of differentially expressed genes (DEGs) in the pairwise comparisons. D0: day 0; D3: day 3; D7: day 7; D14: day 14.

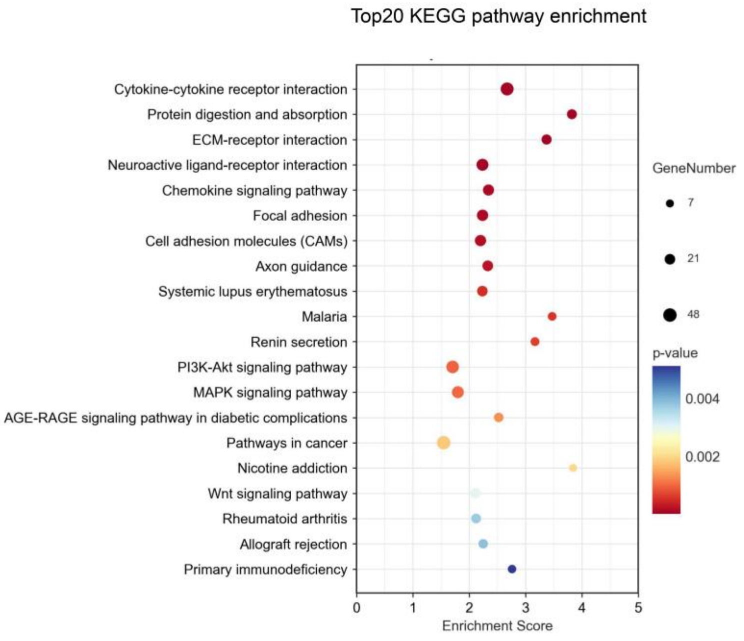

Figure S3. The top 20 enriched Kyoto Encyclopedia of Genes and Genomes (KEGG) pathway terms of differentially expressed genes (DEGs). ECM: extracellular matrix.

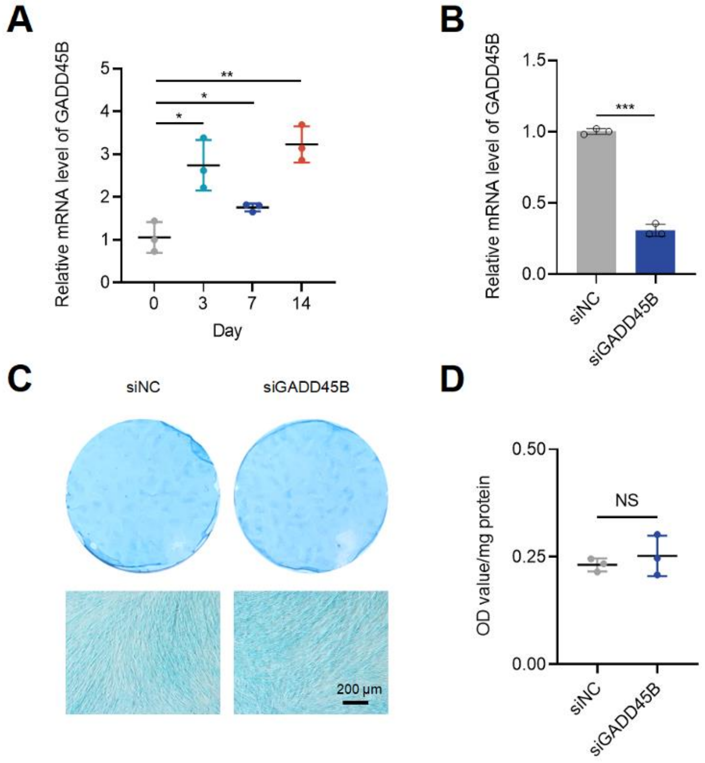

Figure S4. A) RT-qPCR showing the mRNA level of GADD45B during chondrogenic differentiation of DPSCs. B) RT-qPCR analysis showing the knockdown efficiency of siRNA against GADD45B (siGADD45B). C) Alcian blue staining revealing GADD45B knockdown had no significant effect on proteoglycan production after chondrogenic induction for 2 weeks. Scale bar: 200 μm. D) Quantification of proteoglycans synthesis at 2 weeks. Data are presented as mean ± SD (n = 3). Statistical analysis: $*p < 0.05$, $**p < 0.01$, $***p < 0.001$. NS: not significant.

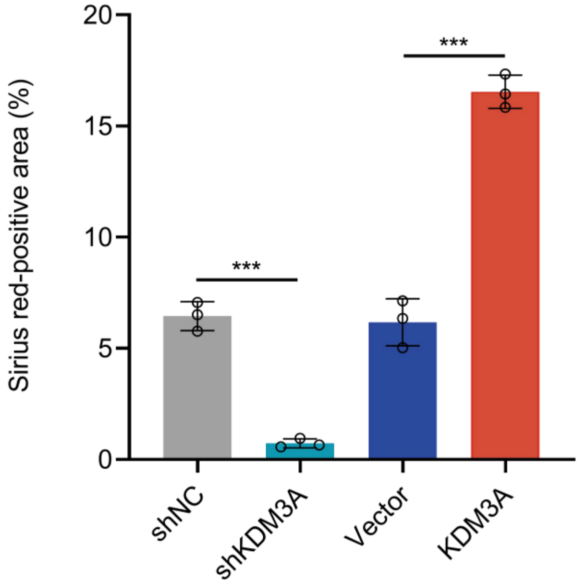

Figure S5. Quantitative analysis of sirius red staining obtained from Figure 3M. Data are presented as mean ± SD (n = 3). Statistical analysis: ***p < 0.001.

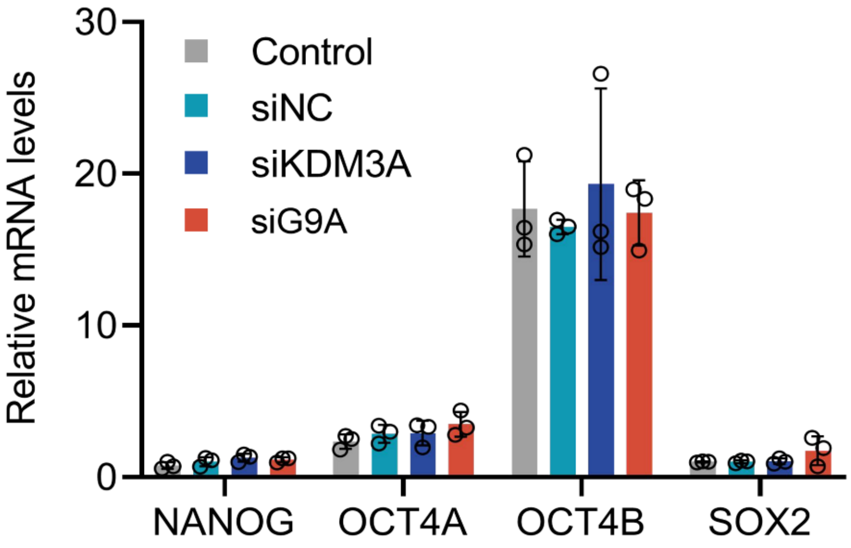

Figure S6. RT-qPCR analysis showing the expression of stem cell markers in DPSCs. Data are presented as mean ± SD (n = 3).

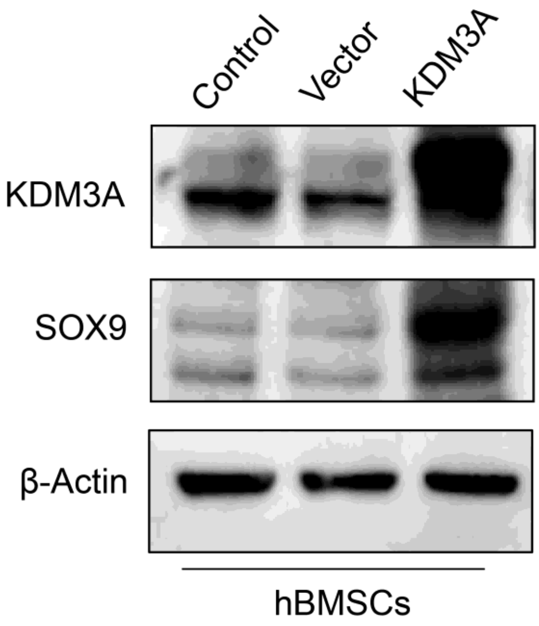

Figure S7. Western blotting indicating enhanced SOX9 protein level after KDM3A overexpression in human bone marrow mesenchymal stem cells (hBMSCs).

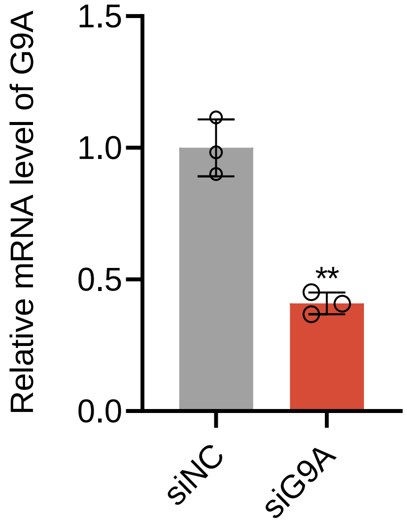

Figure S8. RT-qPCR analysis showing the knockdown efficiency of siRNA against G9A (siG9A). Data are presented as mean ± SD (n = 3). Statistical analysis: $^{**}p < 0.01$.

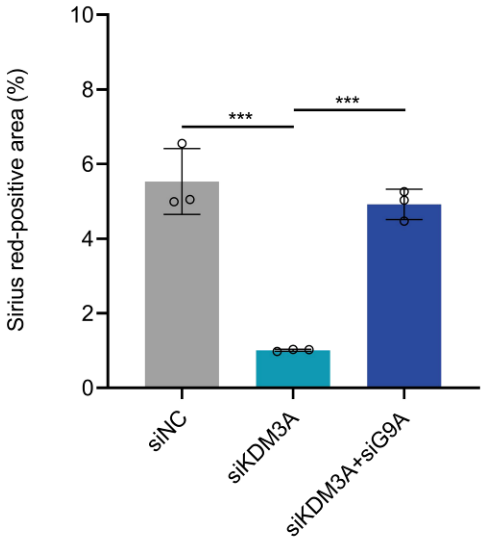

Figure S9. Quantitative analysis of sirius red staining obtained from Figure 4K. Data are presented as mean ± SD (n = 3). Statistical analysis: ***p < 0.001.

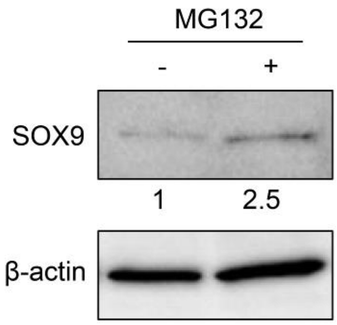

Figure S10. Western blotting revealing enhanced SOX9 protein level after DPSCs incubated with MG132 (20 µM) for 5 h.

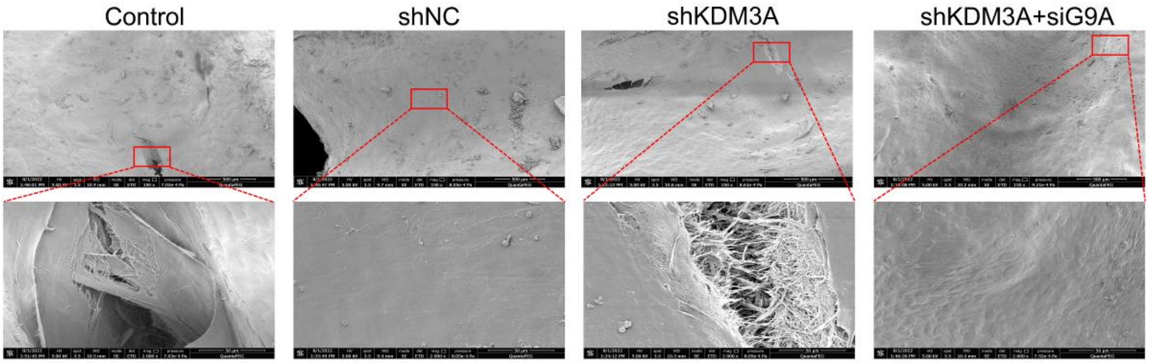

Figure S11. Scanning electron microscopy (SEM) images of the cartilage repair area. Scale bars: 50 µm, 500 µm.

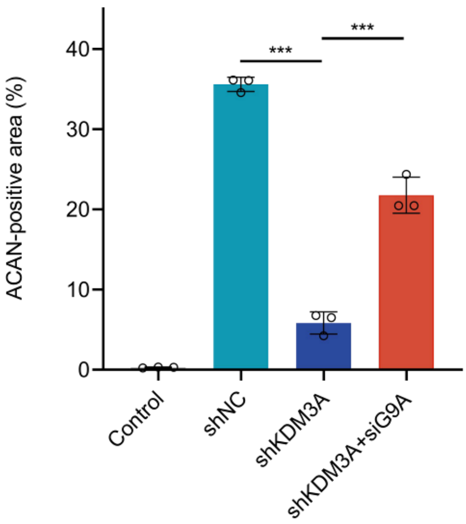

Figure S12. Quantitative analysis of immunofluorescence staining obtained from Figure 7E. Data are presented as mean ± SD (n = 3). Statistical analysis: ***p < 0.001.

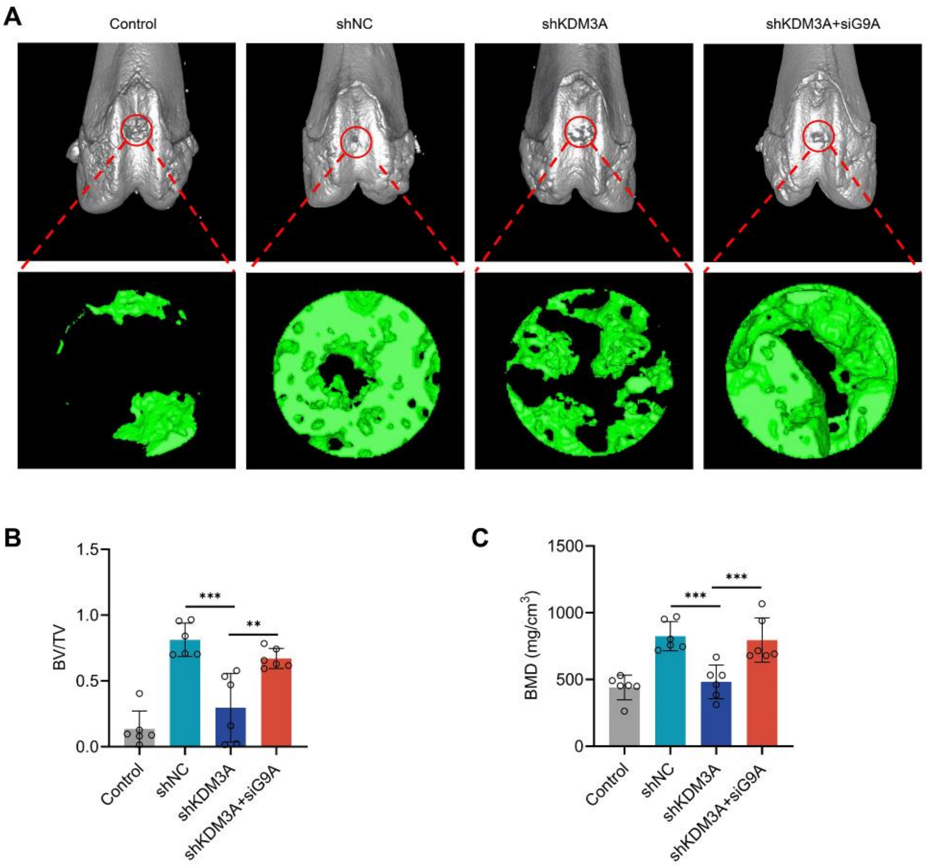

Figure S13. A) 3D reconstruction of μCT images of new subchondral bone formation in rat knee at 8 weeks after DPSCs implantation. B) Quantitative analysis of subchondral volume/tissue volume (BV/TV) of regenerated bone tissue. C) Quantitative analysis of bone mineral density (BMD) of regenerated subchondral bone tissue. Data are presented as mean ± SD (n = 6). Statistical analysis: **p < 0.01, ***p < 0.001.

Reference

[1] P. Wang, Y. Li, T. Meng, J. Zhang, Y. Wei, Z. Meng, Y. Lin, D. Liu, L. Sui, *Cell Prolif.* **2018**, *51*, e12413.

[2] Y. Zeng, R. Qiu, Y. Yang, T. Gao, Y. Zheng, W. Huang, J. Gao, K. Zhang, R. Liu, S. Wang, Y. Hou, W. Yu, S. Leng, D. Feng, W. Liu, X. Zhang, Y. Wang, *Cell Rep.* **2019**, *29*, 1482.

[3] D. Huang, Q. Li, X. Sun, X. Sun, Y. Tang, Y. Qu, D. Liu, T. Yu, G. Li, T. Tong, Y. Zhang, *Cell Death Differ.* **2021**, *28*, 1593.

[4] G. D. Smith, J. Taylor, K. F. Almqvist, C. Erggelet, G. Knutsen, M. Garcia Portabella, T. Smith, J. B. Richardson, *Arthroscopy* **2005**, *21*, 1462.

[5] S. Wakitani, T. Goto, S.J. Pineda, R.G. Young, J.M. Mansour, A.I. Caplan, V. M. Goldberg, *J. Bone Joint Surg. Am.* **1994**, *76*, 579.

[6] J. J. Su, Q. N. Sun, S. Lin, S. Lu, Q. Zhang, Y. X. Li, B. Li, Y. Sun, K. Liu, H. J. Zhang, F. Wang, J. J. Li, Y. Wei, *Adv. Funct. Mater.* **2022**, 2204271.
